# Supplementary material for: INK4a/ARF Expression Impairs Neurogenesis in the Brain of Irradiated Mice
Source: Stem Cell Reports. 2018 Apr 26;10(6):1721–33. doi: 10.1016/j.stemcr.2018.03.025 (PMC5989693; doi:10.1016/j.stemcr.2018.03.025)
Supplement: Document S1. Figures S1–S6 [file mmc1.pdf]

Stem Cell Reports, Volume *10*

Supplemental Information

INK4a/ARF Expression Impairs Neurogenesis in the Brain of Irradiated Mice

Oanh Le, Lina Palacio, Gilbert Bernier, Ines Batinic-Haberle, Gilles Hickson, and Christian Beauséjour

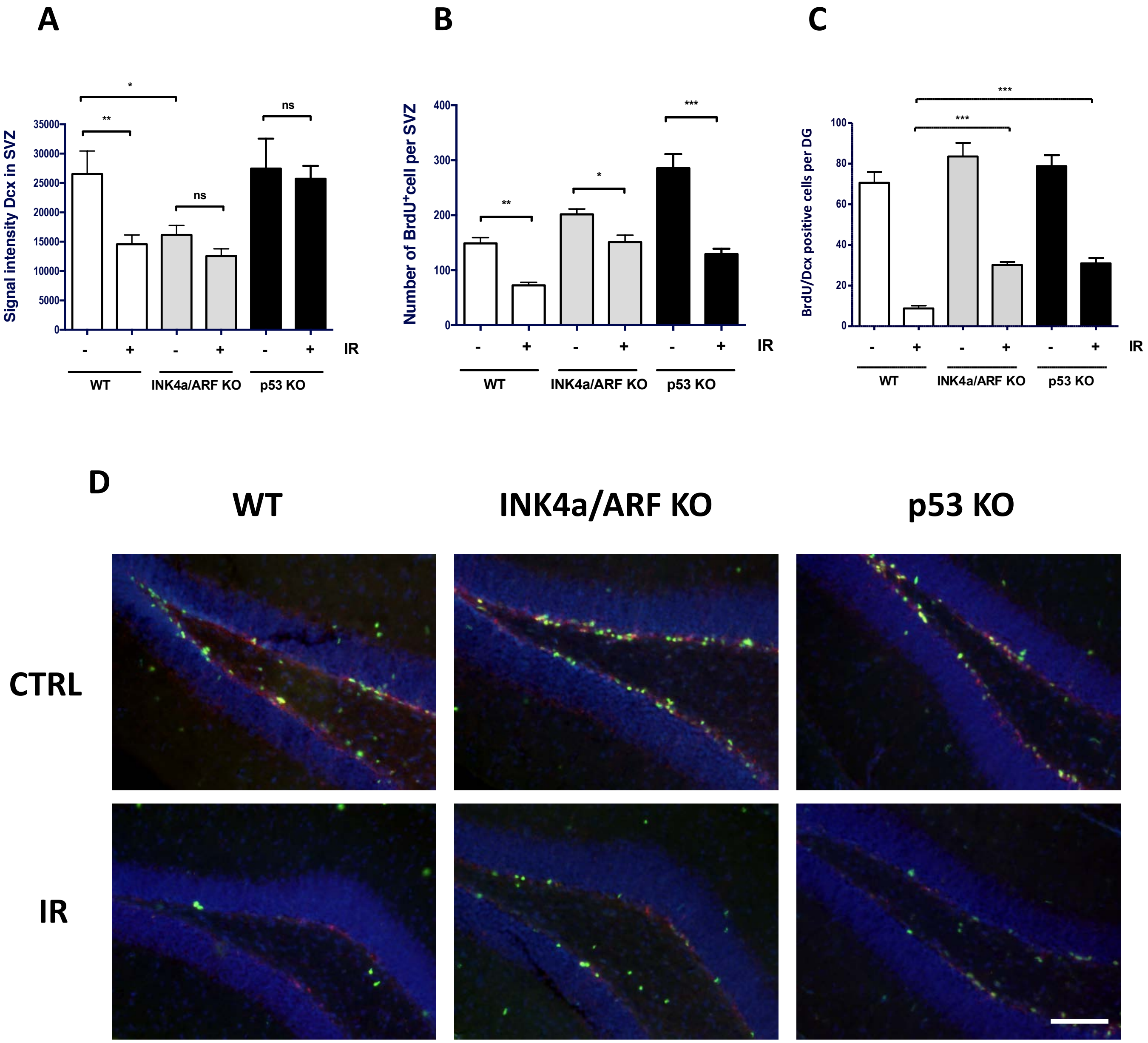

Figure S1. Neurogenesis recovery after IR in absence of INK4a/ARF or p53 expression. (A) Wild-type (WT), *ink4a/arf*-null (INK4a/ARF KO) and *p53*-null (p53 KO) mice were irradiated or not at a dose of 6 Gy and quantification of Dcx signal intensity in the SVZ determined **(B)** Numbers of BrdU positive cells detected in SVZ. **(C)** BrdU incorporation mostly occurs in Dcx positive cells independently of mouse genotypes. Mice were irradiated or not at a dose of 6 Gy and quantification of double positive Dcx/BrdU cells in the DG. **(D)** Representative images showing BrdU incorpotation in Dcx positive cells in the DG. Scale bar = 100 microns. Average from n= 3-6 sections from at least 3 different mice were used. p values were obtained by performing a non parametric ANOVA (Kruskal-Walli) test (***$p<0.001$, **$p<0.01$, *$p<0.05$). ns= no significant difference was observed.

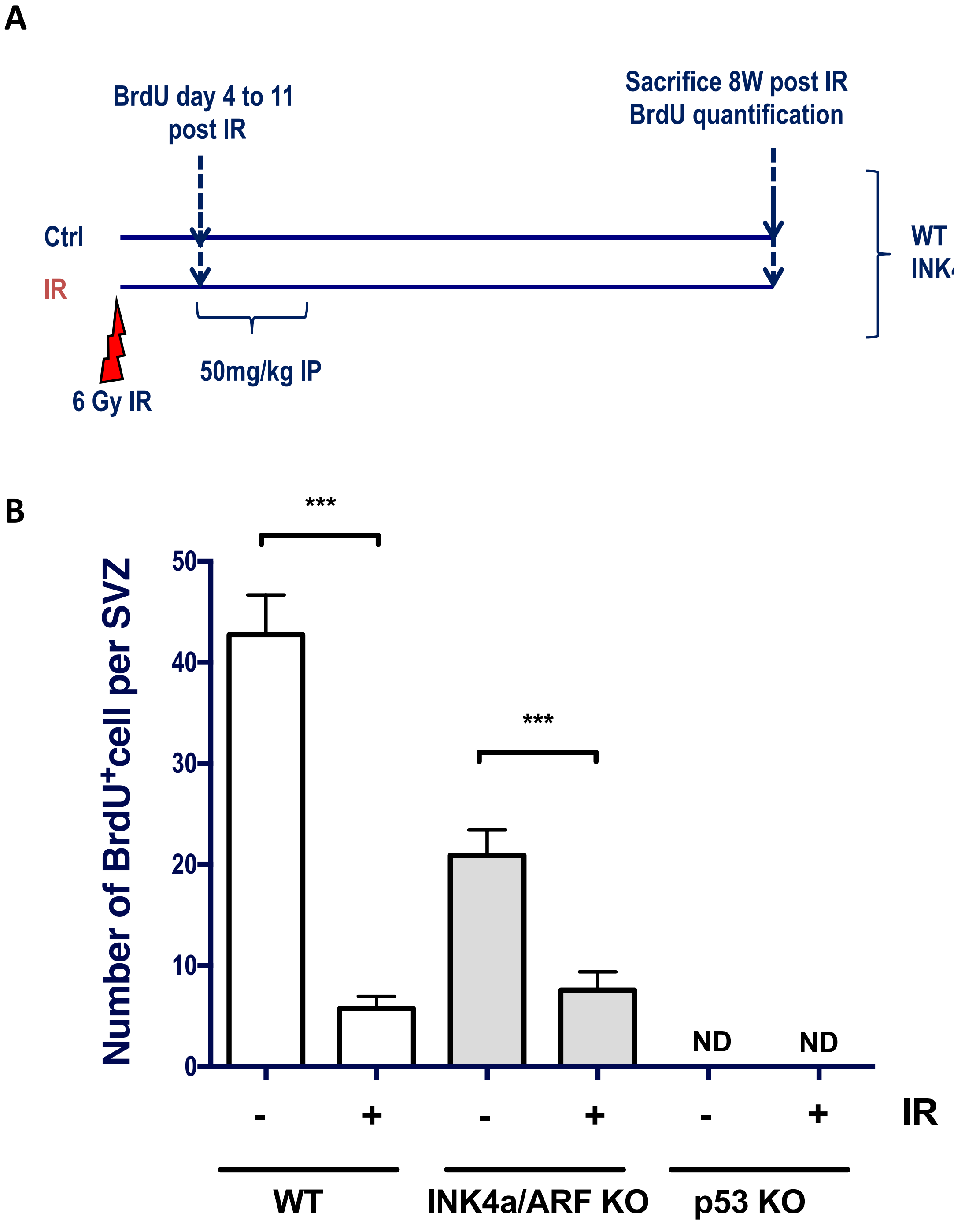

Figure S2. The proportion of BrdU retaining cell long term after IR in WT and INK4a/ARF KO mice. (A) Schematic of the experiment. **(B)** Wild-type (WT) and *ink4a/arf*-null (INK4a/ARF KO) mice were irradiated (6 Gy) and injected intraperitoneally (IP) with BrdU at a dose of 50 mg/kg once a day for 7 days starting on day 4 post exposure to IR. 8 weeks later, mice were sacrificed and the number of BrdU positive cells remaining in the SVZ counted. Shown is the average number of cells per field. n= 6 sections from at least 3 different mice was used. ***p<0.001. nd = not determined.

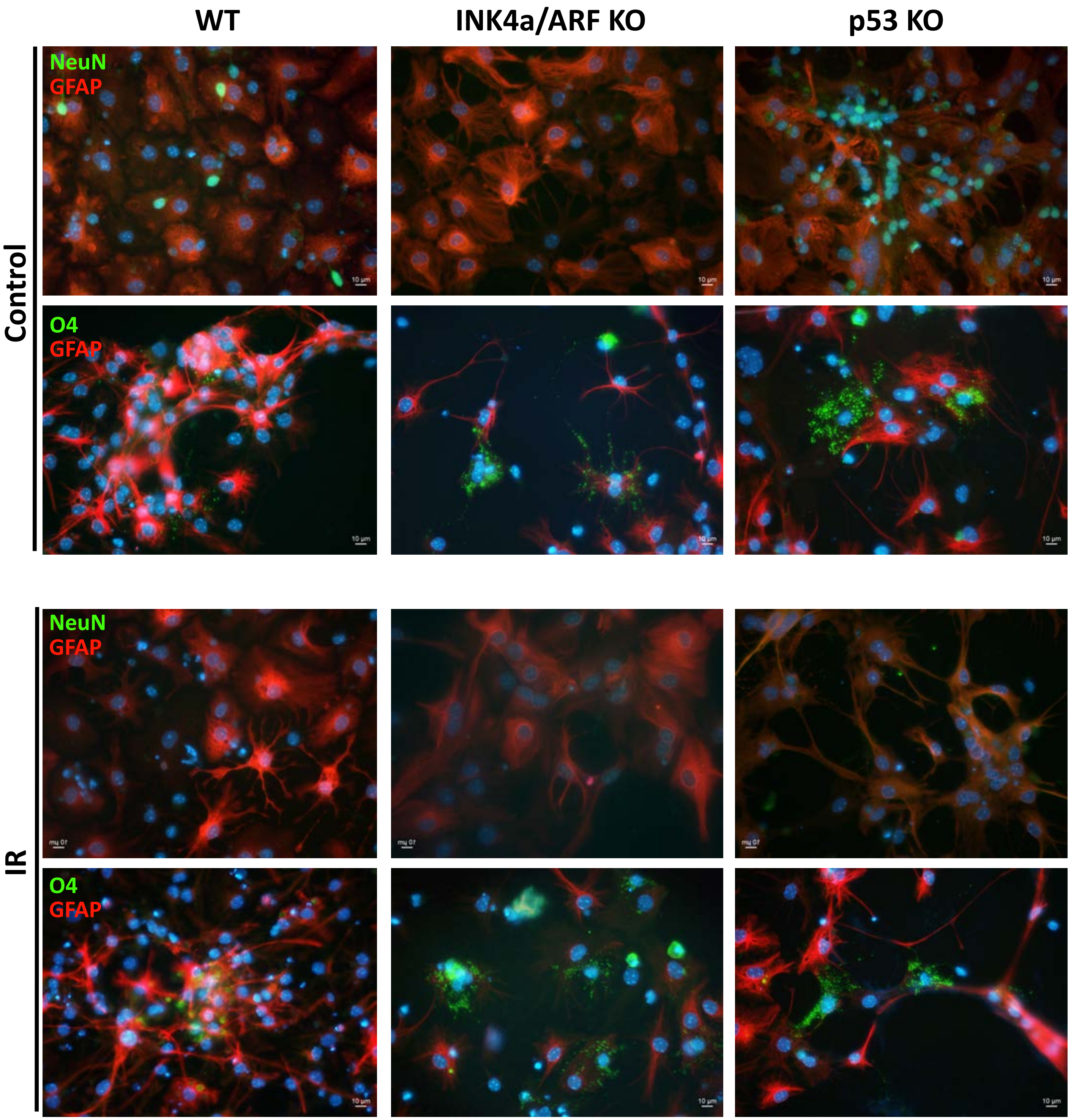

Figure S3. Differentiation potential of secondary neurospheres. Secondary neurospheres from control or irradiated (IR) wild-type (WT), *ink4a/arf*-null (INK4a/ARF KO) and *p53*-null (p53 KO) mice were dissociated, expanded and differentiated into neurons (NeuN in green, upper panel), astrocytes (GFAP in red) or oligodendrocyte (O4 in green, lower panel). Representative images of 2 independent experiments are shown. Nuclei were stained with DAPI (in blue).

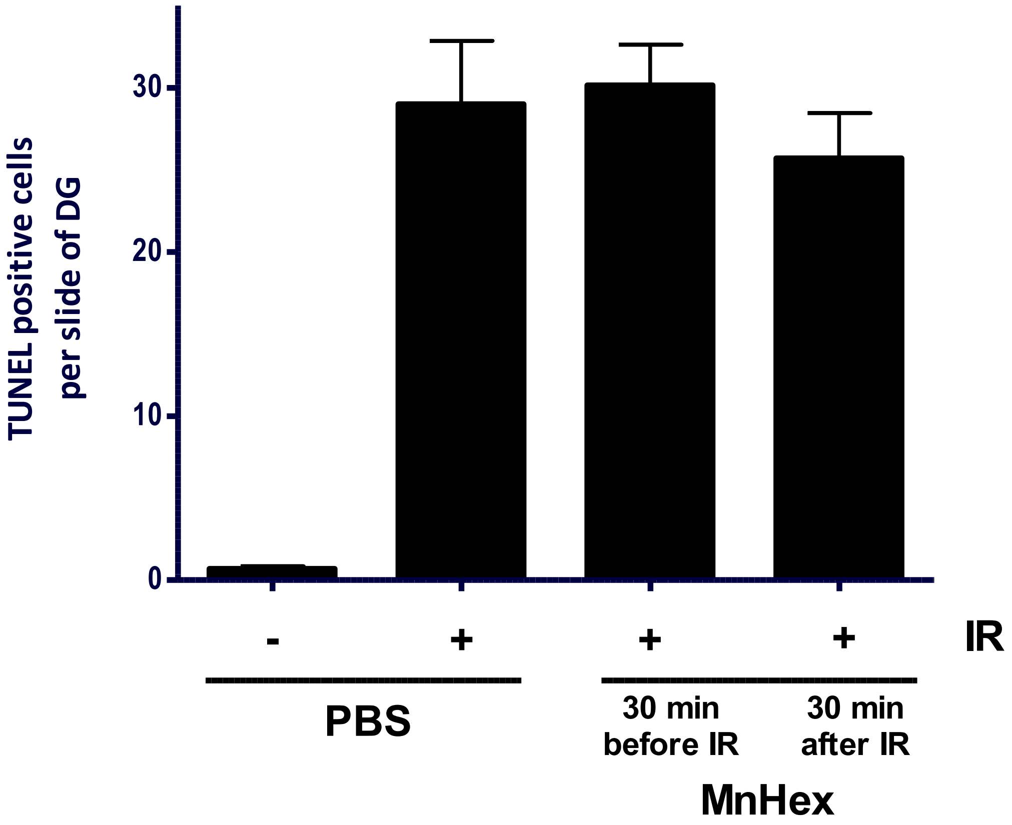

Figure S4. Injection of MnHex does not prevent induction of apoptosis in the irradiated brain. Quantification of the number of apoptotic cells, as detected by TUNEL, in the DG 6 hours following exposure of wild-type (WT) mice injected subcutaneously or not with a single dose of 450 ug/kg of MnHex 30 minutes before or after 6 Gy cranial IR. Indicated is the average number of apoptotic cells per DG section +/- SEM n=4 mice per group.

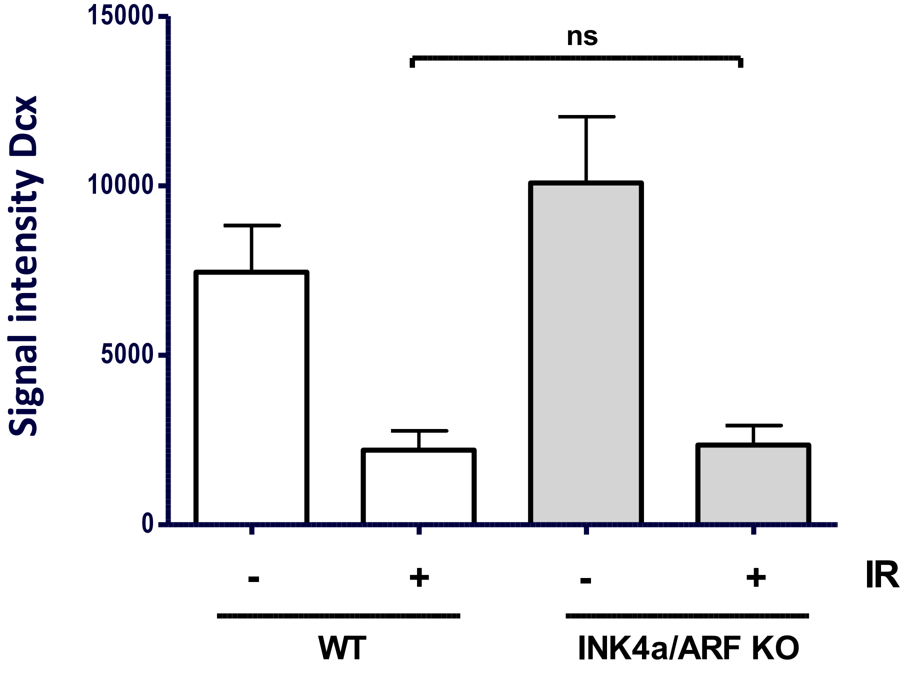

Figure S5. Absence of INK4a/ARF expression does not favors neurogenesis in the DG short term following exposure to IR. Wild-type (WT) and *ink4a/arf*-null (INK4a/ARF KO) mice received or not cranial irradiation at a dose of 6 Gy and quantification of Dcx signal intensity was determined in the DG two weeks after IR. Shown is the average +/- SEM. n= 4 mice per group. NS, no significant difference was observed.

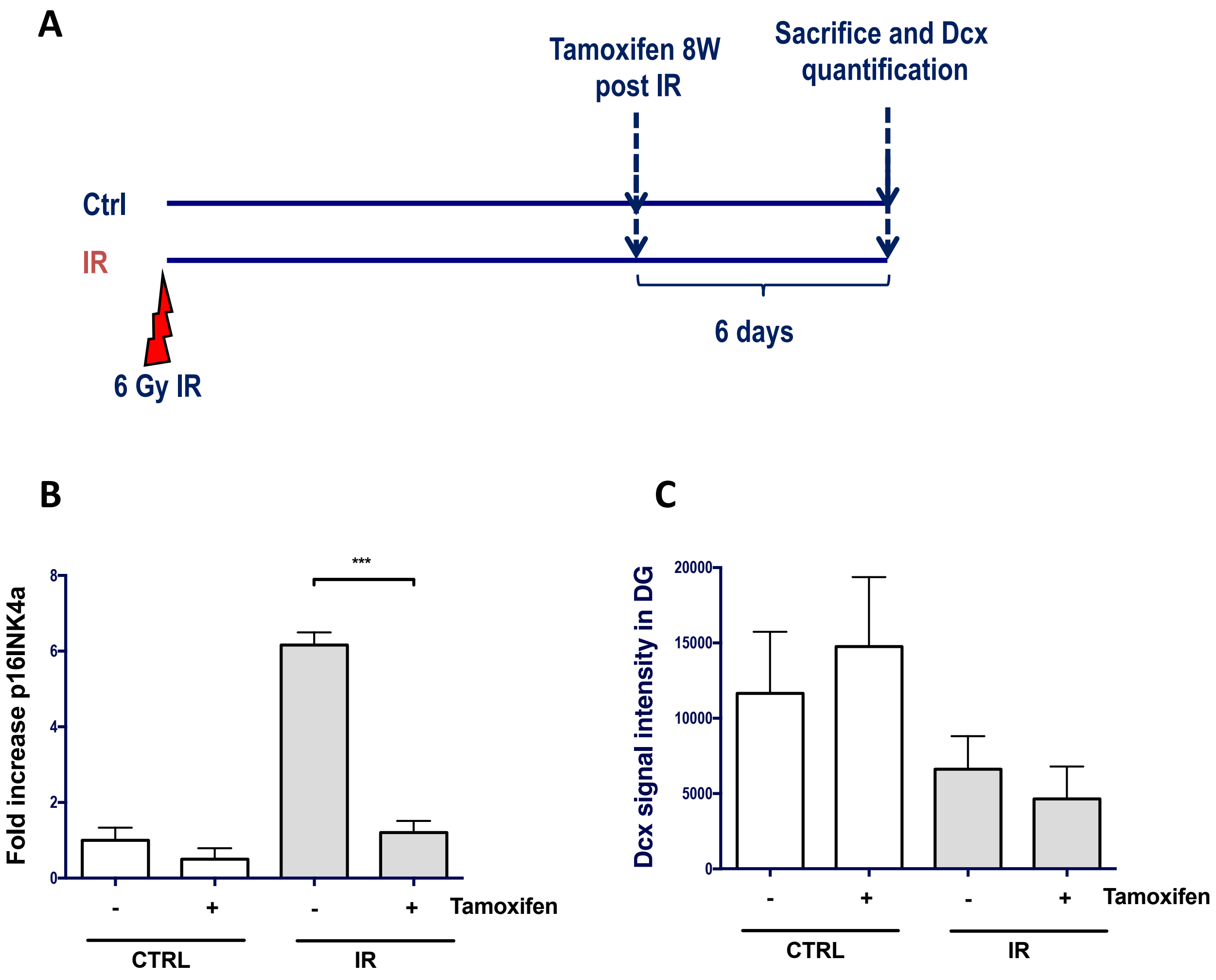

Figure S6. Conditional deletion of p16INK4a does not increase neurogenesis in the DG following exposure to IR. We used conditional *p16INK4a*-null mice in which p16INK4a expression is lost upon the injection of tamoxifen (Cre-ERT^2 recombinase under the human ubiquitin C promoter). **(A)** Schematic of the experiment. 8 weeks after irradiation, mice were treated or not with tamoxifen at a dose of 200 mg/kg (diluted in a mixture 1:50 of ethanol and corn oil respectively – see Palacio et al. Oncogene 2016) by gavage for 5 consecutive days. Mice were sacrificed on day 6 following the first injection of tamoxifen. **(B)** RNA was extracted from the brain and expression of p16INK4a determined by quantitative real-time PCR and normalized to 18S. **(C)** Quantification of Dcx staining was determined in the DG and signal intensity adjusted to the size of the DG on each section. Shown is the average +/- SEM from n= 10-12 sections collected from 4 different mice per group. ***p<0.001.
