# Supplementary material for: INK4a/ARF Expression Impairs Neurogenesis in the Brain of Irradiated Mice
Source: Stem Cell Reports. 2018 Apr 26;10(6):1721–33. doi: 10.1016/j.stemcr.2018.03.025 (PMC5989693; doi:10.1016/j.stemcr.2018.03.025)
Supplement: Document S2. Article plus Supplemental Information [file mmc2.pdf]

Stem Cell Reports
Article

OPEN ACCESS

INK4a/ARF Expression Impairs Neurogenesis in the Brain of Irradiated Mice

Oanh Le,[1] Lina Palacio,[1,5] Gilbert Bernier,[2] Ines Batinic-Haberle,[3] Gilles Hickson,[1,4] and Christian Beauséjour[1,5,*]
[1]Centre de Recherche du CHU Ste-Justine, 3175 Côte Sainte-Catherine, Montréal, Québec H3T 1C5, Canada
[2]Centre de Recherche de l'Hôpital Maisonneuve Rosemont and Department of Ophtalmology, Université de Montréal, Montréal, Québec, Canada
[3]Department of Radiation Oncology-Cancer Biology, Duke University, Duke Cancer Center, Medicine Circle, Durham, NC 27710, USA
[4]Department of Pathology and Cell Biology, Université de Montréal, Montréal, Québec, Canada
[5]Department of Pharmacology and Physiology, Université de Montréal, Montréal, Québec, Canada
*Correspondence: c.beausejour@umontreal.ca
https://doi.org/10.1016/j.stemcr.2018.03.025

SUMMARY

Brain neurogenesis is severely impaired following exposure to ionizing radiation (IR). We and others have shown that the expression of the tumor suppressor gene *p16INK4a* is increased in tissues exposed to IR and thus hypothesized that its expression could limit neurogenesis in the irradiated brain. Here, we found that exposure to IR leads to persistent DNA damage and the expression of p16INK4a in the hippocampus and subventricular zone regions. This was accompanied by a decline in neurogenesis, as determined by doublecortin expression and bromodeoxyuridine incorporation, an effect partially restored in *Ink4a/arf*-null mice. Increased neurogenesis in the absence of INK4a/ARF expression was independent of apoptosis and activation of the microglia. Moreover, treatment of irradiated mice with a superoxide dismutase mimetic or clearance of p16INK4a-expressing cells using mouse genetics failed to increase neurogenesis. In conclusion, our results suggest that IR-induced p16INK4a expression is a mechanism that limits neurogenesis.

INTRODUCTION

Exposure of the brain to ionizing radiation (IR) is associated with impaired memory and learning deficits, a phenotype often observed in cancer survivors (Krull et al., 2013; Robison and Hudson, 2014). Exposure to IR also leads to a drastic loss in neuronal progenitor cell counts, in a dose-dependent manner (Lu and Wong, 2007; Monje et al., 2002; Tada et al., 1999). Postnatal neurogenesis is limited to the dentate gyrus (DG) of the hippocampus and the subventricular zone (SVZ)/olfactory bulb regions. Neurogenesis is often defined experimentally by the incorporation of a tracer compound such as bromodeoxyuridine (BrdU) and the labeling of young neurons with doublecortin (DCX) (Wojtowicz, 2006). Loss in neurogenesis appears to be permanent in the subgranular zone of the DG, while it was shown to recover, at least partially, in the SVZ of the lateral ventricle (Hellstrom et al., 2009, 2011). Furthermore, evidence suggests that loss of hippocampal neurogenesis is strongly correlated with cognitive impairment (Monje and Dietrich, 2012; Rao et al., 2011; Rola et al., 2004).

Despite numerous important side effects, radiotherapy is still arguably one of the most effective tools in the treatment of cancer. In this context, it is essential to understand the mechanisms by which radiotherapy limits neurogenesis in the long term. IR-induced apoptosis of neural stem and progenitor cells is one mechanism that can lead to loss of neurogenesis (Limoli et al., 2004; Mizumatsu et al., 2003). IR-induced inflammation was also shown to compromise neurogenesis, presumably by altering the neurogenic niche through, for example, the secretion of cytokines by the activated microglia (Ekdahl et al., 2003; Lee et al., 2013; Monje et al., 2003; Moravan et al., 2011). Coincidently, generation of reactive oxygen species (ROS) following exposure to IR also impairs neurogenesis, an effect that can be attenuated by treatment with antioxidant enzymes and metabolites (Acharya et al., 2010; Zou et al., 2012).

Precisely how neurogenesis is impaired in the long term following IR is unclear. One possibility is that neuronal progenitor cells are mostly all eliminated following radiotherapy-induced p53-dependent apoptosis. Alternatively, IR-induced inflammation and ROS may cause deleterious modification to the neuronal niche, which could restrict progenitor cell differentiation and proliferation. Another possibility is that DNA damage and oxidative stress, by inducing an INK4a/ARF-dependent growth arrest, may interfere with neurogenesis. Indeed, we and others have shown the expression of p16INK4a, a robust senescence/aging marker, and to a lesser extent p19ARF, is increased in murine and human tissues exposed to IR (Krishnamurthy et al., 2004; Le et al., 2010; Marcoux et al., 2013). Thus, while an increase in INK4a/ARF expression prevents damaged cells from proliferating further, it might also prevent the regenerative potential of irradiated tissues by inducing stem/progenitor cells senescence (van Deursen, 2014). Remarkably, *INK4a-null* mice exhibit improved regenerative potential in several organs with age, hence suggesting that the accumulation of senescent stem/progenitor cells is deleterious for the organism (Krishnamurthy et al., 2006; Molofsky et al., 2006). For

This is an open access article under the CC BY license (http://creativecommons.org/licenses/by/4.0/).

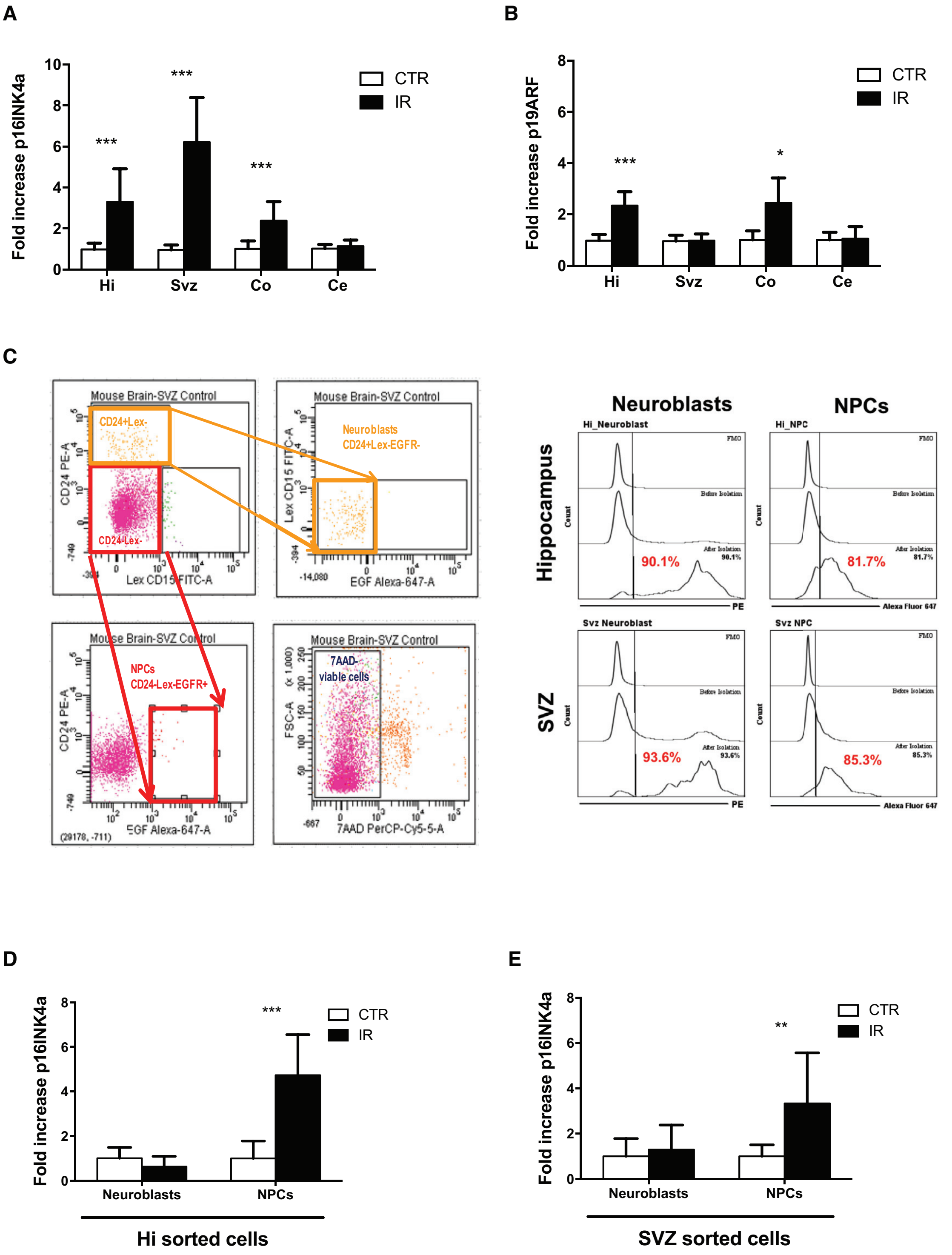

Figure 1. INK4a/ARF Expression in Selectively Induced Irradiated Brain Cells and Regions
(A and B) Mice were exposed or not to 6 Gy cranial radiation, and 8–12 weeks later RNA was extracted from the hippocampus (Hi), subventricular zone (Svz), cortex (Co), and cerebellum (Ce). Expression of p16INK4a (A) and p19ARF (B) as determined by real-time qPCR and normalized to 18S.

(legend continued on next page)

example, the ability to form new neurons was shown to decline in the SVZ of aged mice brains, a defect that was partially abrogated in mice deficient in p16INK4a (Molofsky et al., 2006).

In this study we provide evidence that IR-induced INK4a/ARF expression is a mechanism by which loss of brain neurogenesis occurs. Moreover, we also found this effect to be likely cell-autonomous and independent from apoptosis or activation of the microglia.

RESULTS

INK4a/ARF Expression Is Induced in Selected Brain Regions Following Exposure to IR

We previously showed that p16INK4a and, to a lesser extent p19ARF, are expressed in a delayed manner (8–12 weeks) in various mouse tissues following exposure to IR (Le et al., 2010). The reason for such a delay in expression is unknown but may reflect the need for cells to persist in tissues for several weeks following DNA damage or to attempt cell division, two criteria fulfilled by progenitor/stem cells. This is supported by the observation that hematopoietic stem cells, but not their progeny, have an increase in p16INK4a expression in the weeks following their exposure to IR (Wang et al., 2006). We thus hypothesized that INK4a/ARF expression would be higher in irradiated brain regions enriched in neuronal progenitor cells. As expected, 8–12 weeks post exposure to 6 Gy cranial IR, we found that p16INK4a expression was increased in the hippocampus and the SVZ compared with the same tissues isolated from age-matched non-irradiated mice (Figure 1A). Surprisingly, expression of p16INK4a was also found elevated in the cortex while it was not in the cerebellum. Conversely, p19ARF expression was found increased only in the hippocampus and cortex regions (Figure 1B). Moreover, when cells from the hippocampus or the SVZ were sorted based on specific cell markers (CD24+/LEX−/EGFR− for neuroblasts and CD24−/LEX−/EGFR+ for NPCs), we found distinct expression profiles in these populations (Figures 1C–1E). For example, in both regions, p16INK4a expression was increased in NPCs but not in neuroblasts. These observations are in line with previous results showing that INK4a/ARF expression is preferentially increased in progenitor cell populations isolated from muscle, fat or bone (Baker et al., 2013; Despars et al., 2013).

Absence of INK4a/ARF Expression Favors Neurogenesis in the Irradiated Brain

Whether an increase in INK4a/ARF expression contributes to the loss of brain neurogenesis observed following exposure to IR is unknown. To answer this question, *Ink4a/arf*-null and wild-type mice were exposed or not to 6 Gy cranial radiation. Mice were allowed to recover for 8 weeks and then injected with BrdU for 10 consecutive days prior to sacrifice (Figure 2A). We chose to sacrifice mice no longer than 8 weeks post IR because of the cancer susceptibility of *Ink4a/arf*-null mice (Sharpless et al., 2001).

We first evaluated neurogenesis by measuring the expression of DCX, a marker of newborn neurons, on free-floating brain sections. We found a significantly higher level of new neurons being formed in the DG of irradiated *Ink4a/arf-null* compared with wild-type mice, where DCX expression was almost completely absent (Figures 2B and 2D). The intensity of the DCX signal measured in the absence of INK4a/ARF expression was slightly lower than that observed in the absence of apoptosis in the irradiated brains of *p53-null* mice (Figures 2B and 2D). *p53-null* mice were used here as a comparison to evaluate how effective INK4a/ARF deletion is in protecting mice against loss of neurogenesis. Similar to that observed in the DG, the absence of INK4a/ARF or p53 expression also resulted in an increased DCX signal in the irradiated SVZ region (Figure S1A). However, we found IR-induced loss of neurogenesis was less severe in the SVZ compared with the DG (Figure S1A). This likely explains why the absence of INK4a/ARF or p53 expression allowed almost full neurogenesis recovery in the SVZ. Of note, we consistently observed lower levels of new neurons (DCX+) cells in the SVZ of *Ink4a/arf-null* mice compared with wild-type or *p53-null* mice (Figure S1A).

To more closely evaluate the impact of INK4a/ARF expression on neurogenesis, we also measured the incorporation of BrdU in the DG and SVZ 8 weeks post exposure to IR. As for DCX expression, we observed a much higher level of BrdU+ cells in the irradiated DG of *Ink4a/arf-null* compared with wild-type mice in which BrdU incorporation was found almost completely inhibited (Figures 2C, 2D, and S1B). Again, the increase in BrdU incorporation in the absence of INK4a/ARF was similar to that observed in *p53-null* mice (Figures 2C and 2D). Moreover, the majority of BrdU+ cells identified were at the inner edge of the DG subgranular zone region and found to also express the DCX marker (Figures S1C and S1D). However, a BrdU pulse performed early after IR (day 4–11) suggested that

(C–E) SVZ (as shown) or hippocampus regions were dissociated and viable (7AAD−) cells populations (CD24+/LEX−/EGFR− for neuroblasts in orange and CD24−/LEX−/EGFR+ for NPCs in red) were sorted by fluorescence-activated cell sorting. (C) Purity of the sorted cell populations was determined by flow cytometry. RNA was then extracted and p16INK4a expression determined in neuroblasts and NPCs populations from the Hi (D) or the SVZ (E). n = 4–10 mice per group.

$^{*}p < 0.05$, $^{**}p < 0.01$, $^{***}p < 0.001$, obtained by performing a Student's t test.

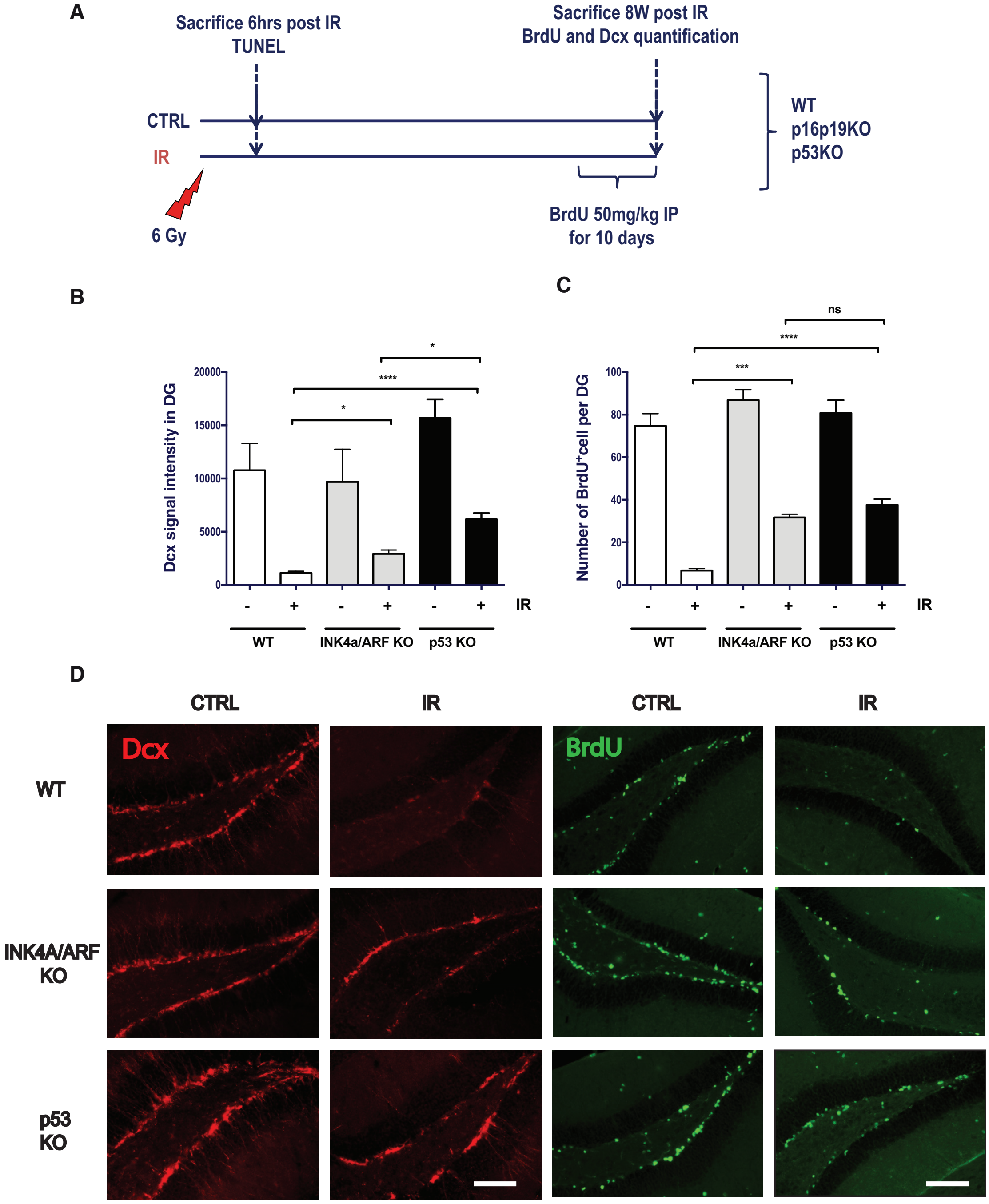

Figure 2. Absence of INK4a/ARF Expression Favors Neurogenesis in the Irradiated Hippocampus

(A) Schematic of the experiments. Wild-type (WT), *ink4a/arf*-deficient (INK4a/ARF knockout [KO]) and *p53*-deficient (p53 KO) mice were irradiated or not at a dose of 6 Gy and injected with BrdU 10 days prior to sacrifice.

(B) Quantification of DCX staining was determined in the DG and signal intensity adjusted to the size of the DG on each section.

(C) The number of BrdU+ cells in the DG was determined and counts adjusted to the size of the DG on each section. n = 3–4 mice per group, with a minimum of two sections per mouse analyzed.

(D) Representative images showing DCX expression and incorporation of BrdU in the DG. The p values were obtained by performing a non-parametric ANOVA (Kruskal-Wallis) test. *$p < 0.05$; ***$p < 0.001$; ****$p < 0.0001$; ns, no significant difference was observed. Scale bars, 100 μm.

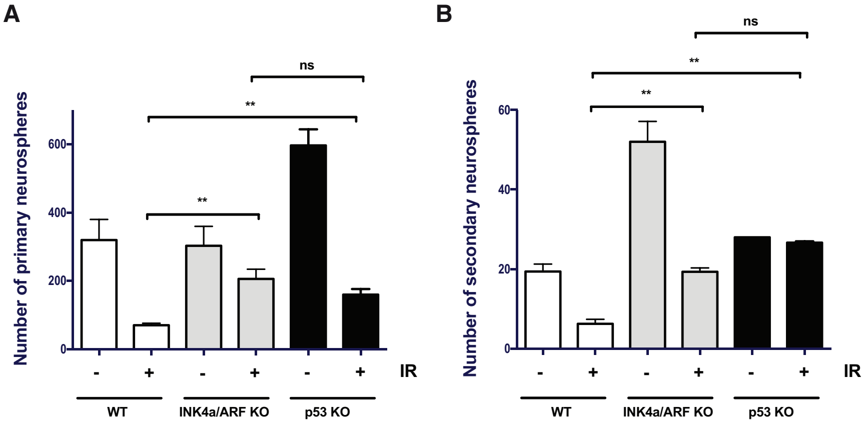

C

	WT	INK4a/ARF KO	P53 KO	WT	INK4a/ARF KO	P53 KO
	control			IR		
Astrocytes (GFAP⁺)	+++	+++	+++	+++	+++	+++
Oligodendrocytes (O4⁺)	+	++	++	+	++	++
Neurons (NeuN⁺)	+	-	++	-	-	-

Figure 3. Increased Neuronal Precursor Cells Counts Following Exposure to IR in Absence of INK4a/ARF Expression

(A) Eight weeks following exposure of WT, *ink4a/arf*-null (INK4a/ARF KO) and *p53*-null (p53 KO) mice to 6 Gy cranial IR, cells from the SVZ were dissociated and neurospheres were counted from n = 4 to 10 mice per group.

(B) Primary neurospheres from (A) were dissociated and secondary neurospheres were then counted manually using an inverted microscope 7 days later. The number of neurospheres per field from n = 3 to 6 primary cultures is shown.

(C) Secondary neurospheres were dissociated, expended, and differentiated in NeuroCult differentiation media (see Experimental Procedures for details). Seven to 10 days later, coverslips containing cells were removed and fixed, and their differentiation into neurons, oligodendrocytes, and astrocytes was assessed by immunofluorescence. The ability to differentiate (+++, ++, or +) or not (−) in each three cell types is shown. The differentiation procedure was performed twice using two different sets of secondary neurospheres with similar results. The p values (**$p < 0.01$) were obtained by performing a non-parametric ANOVA (Kruskal-Walli) test. ns, no significant difference was observed.

the proliferation of neuronal progenitor cells is not affected in irradiated wild-type versus *Ink4a/arf-null* mice (Figure S2). This was not surprising given that p16INK4a expression is observed only several weeks after IR. Instead, we believe that a limited number of neuronal progenitor cells survive the radiation and that, in the absence of INK4a/ARF expression, they are then allowed to better proliferate, as suggested by the fewer cells retaining BrdU in absence of IR (Figure S2).

Finally, we also wanted to determine if IR-induced INK4a/ARF expression had an impact on the number of the neuronal progenitor cells with the capacity to form neurospheres *in vitro*. In brief, single-cell populations were first isolated from the SVZ region collected before and 8 weeks after mice were exposed to IR and then cultured until distinct neurospheres could be identified. Given the age of our mice at the time of sacrifice (14 weeks), a very limited number of neurospheres was obtained from the DG and thus this region was not used in the study. Neurosphere-forming ability is known to be drastically and permanently reduced after exposure to IR (Lu and Wong, 2007). In line with our results, we observed that the number of primary and secondary neurospheres formed following exposure of mice to IR was significantly higher in the absence of INK4a/ARF expression (Figures 3A and 3B). The absence of p53 expression also allowed higher neurosphere counts at a level similar to what we observed in the absence of INK4a/ARF expression (Figures 3A and 3B). Surprisingly, neurospheres derived from irradiated mice, independently of their genotypes, had lost the ability to differentiate into neurons while they had preserved their ability to differentiate in astrocytes (predominantly) and, to a lesser extent, in oligodendrocytes (Figures 3C and S3). Similarly, neurospheres derived from non-irradiated *INK4a/arf-null* mice were also unable to differentiate in neurons (Figures 3C and S3). The reasons for this are unknown but suggest that the genotoxic stress imposed by the IR and culture conditions favor gliogenesis and/or hamper neuronal differentiation, a phenotype that is amplified in absence of INK4a/ARF.

Absence of p53 but Not INK4a/ARF Expression Prevents IR-Induced Apoptosis and Microglial Activation

To further delineate the mechanism leading to increase neurogenesis, we first determined if INK4a/ARF expression

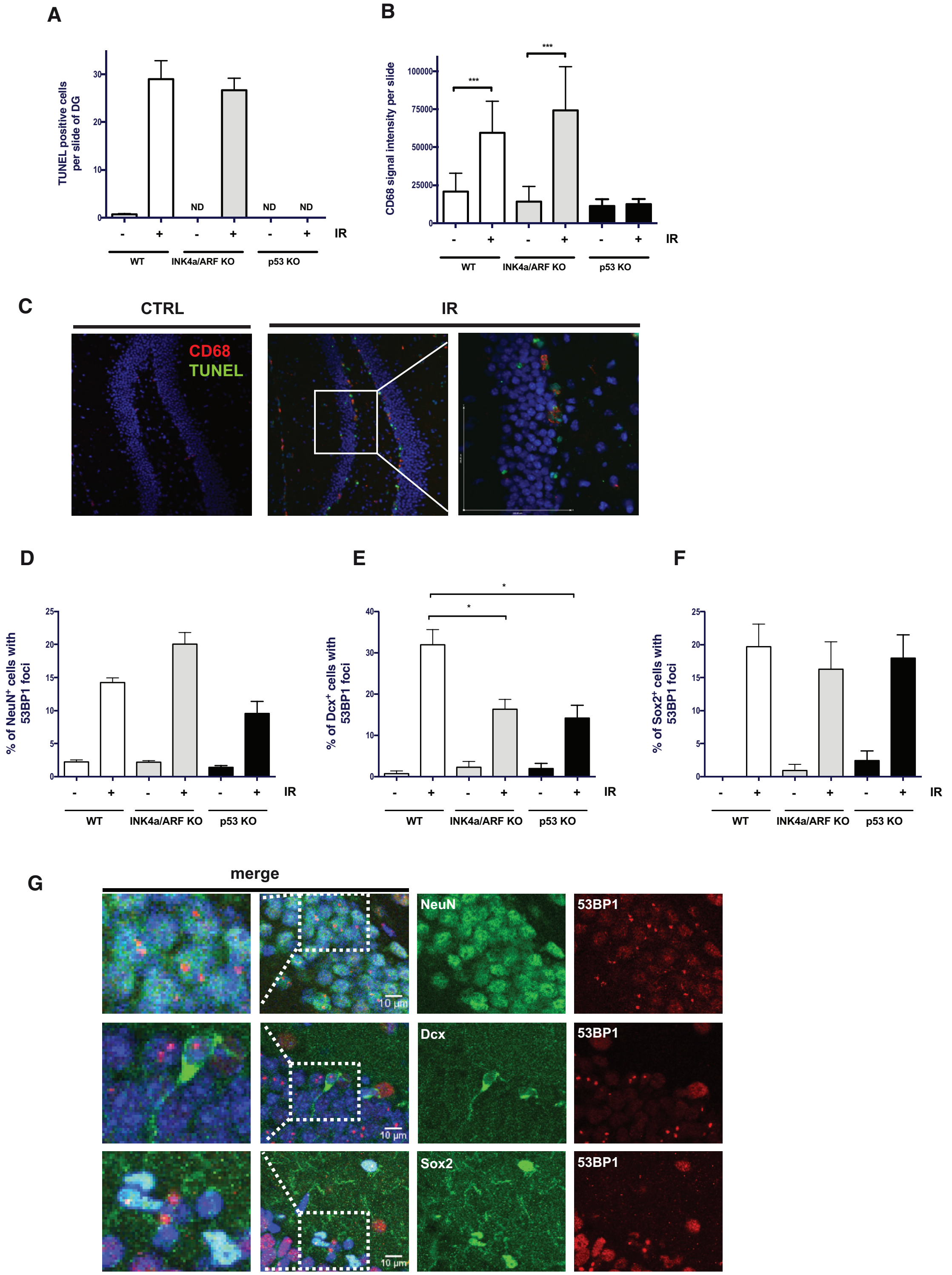

Figure 4. Increased Neurogenesis in the Absence of INK4a/ARF Expression Is Independent of Apoptosis and Microglial Activation
(A) Quantification of the number of apoptotic cells, as detected by TUNEL, in the DG 6 hr following exposure of WT, *ink4a/arf*-deficient (INK4a/ARF KO), and *p53*-deficient (p53 KO) mice to 6 Gy cranial IR. Indicated is the average number of apoptotic cells per DG section (n = 4 mice per group).
(B) Quantification of CD68 expression in the DG 24 hr following exposure of mice to IR.

(legend continued on next page)

had an impact on IR-induced apoptosis. TUNEL immunostaining performed 6 hr following irradiation of the DG revealed that, unlike absence of p53, lack of INK4a/ARF expression conferred no protection against IR-induced apoptosis (Figure 4A). We also noticed that the absence of INK4a/ARF expression did not prevent IR-induced activation of the microglia, as measured using immunostaining against CD68 (Figure 4B). In our model, we found that activation of the microglia occurred only within the first few days post IR and was likely dependent on the presence of apoptotic cells as no activation was measured in the absence of p53 expression (Figures 4B and 4C). These results suggest that lack of INK4a/ARF expression favors neurogenesis independently of apoptosis and activation of the microglia. In parallel, we also observed persistent DNA damage in the hippocampus of irradiated wild-type, *Ink4a/arf*, and *p53-null* mice. Using tissue sections from the DG collected from previously (8 weeks) irradiated mice from all three genotypes, we found the increase in the number 53BP1 foci to be similar in neurons (NeuN+) and progenitor (Sox2+) cells (Figures 4D–4G). However, a significantly lower number of foci were observed in newly formed neurons (DCX+) from *ink4a/arf* and *p53*-null mice compared with wild-type (Figures 4D–4G). These results are in line with the increased neurogenesis observed in these mice.

Treatment with a Superoxide Dismutase Mimetic Reduces IR-Induced Loss of Neuronal Progenitor Cells Growth *Ex Vivo* but Not *In Vivo*

Expression of INK4a/ARF is delayed following exposure to IR, suggesting that it does not increase as a direct effect but rather as a consequence of IR, for example following replicative exhaustion or in response to increased levels of ROS (Ito et al., 2004, 2006). In this context, we hypothesized that treatment of mice with a porphyrin-based superoxide dismutase mimetic, Mn(III) meso-tetrakis (N-n-hexylpyridinium-2-yl) porphyrin, (MnTnHex-2-PyP^{5+}, abbreviated as MnHex), may help prevent loss of neurogenesis (Batinic-Haberle et al., 2014, 2015; Wang et al., 2010). Mice were injected immediately after irradiation with MnHex for eight consecutive weeks until sacrifice. We found that the injection of MnHex could limit p16INK4a expression in the irradiated hippocampus but not in the SVZ (Figures 5A and 5B). Such a reduction in p16INK4a expression did not lead to an increase in DCX signal intensity in the hippocampus (Figure 5C). However, we observed a higher number of neurospheres in mice treated with MnHex compared with saline alone (Figure 5D). The injection of MnHex either before or immediately after exposure to IR did not prevent induction of apoptosis (Figure S4). This effect of MnHex appears independent of p16INK4a as it did not prevent IR-induced p16INK4a expression in the SVZ. However, because MnHex was ineffective at increasing neurosphere counts in irradiated *ink4a/arf-null* mice (Figure 5D), it also implies that the effect MnHex is dependent on INK4A/ARF expression, but perhaps only in a subset of progenitor cells. Of note, the injection of another ROS scavanger, N-acetycysteine (NAC), with a lower bioavailability to the brain compared with MnHex (Giustarini et al., 2012; Weitner et al., 2013), had no effect on p16INK4a expression (data not shown).

Clearance of p16INK4a-Expressing Cells Does Not Restore Neurogenesis

Accumulation of p16INK4a-expressing cells was recently shown to contribute to the development of various pathologies and aging (Baker et al., 2016; Oubaha et al., 2016; Schafer et al., 2017). Hence, we next wanted to explore the possibility that p16INK4a-expressing cells could, for example through an effect on the microenvironment, have an impact on neurogenesis. To this end, we used p16-3MR transgenic mice, which express under the endogenous p16INK4a promoter the *Renilla* luciferase and the herpes simplex virus thymidine kinase genes, the latter metabolizing ganciclovir (GCV) into a toxic drug (Demaria et al., 2014). These mice can selectively eliminate p16INK4a-expressing cells following the injection of GCV. We first confirmed that treatment with GCV allowed for a significant decrease in p16INK4a expression in both the hippocampus and SVZ regions (Figures 6A and 6B). Yet, 10 days following the last injection of GCV no increase in neurogenesis, as measured by DCX staining, was observed in these regions (Figures 6C and 6D). This suggests that the accumulation of p16INK4a-expressing cells does not interfere with brain neurogenesis and/or that a substantial fraction of neuronal progenitor cells are themselves cleared by the GCV. Of note, we did not detect an increase in the luminescent signal of irradiated brains

(C) Representative confocal images showing CD68+ cells (in red) next to apoptotic TUNEL+ cells (in green) following exposure of WT mice to IR. Note the increase in CD68 signal and side by side localization of CD68+ and TUNEL+ cells following IR.
(D–F) Proportion of neurons (NeuN, in green), young neurons (DCX, in green), and progenitor cells (Sox2, in green) showing persistent DNA damage foci (53BP1, in red) in the hippocampus of mice sacrificed 8 weeks post exposure to IR. Nuclei were stained with DAPI.
(G) Representative confocal images of brain sections from irradiated WT mice displaying 53BP1 DNA damage foci with the indicated cell markers are shown. Images were acquired by a Leica microsystems TSC SP8 HyVolution confocal microscope and an HC PL APO CS2 40×/1.3 oil objective. The p values (*p < 0.05; ***p < 0.001) were obtained by performing a Student's t test. ND (no cells were detected).

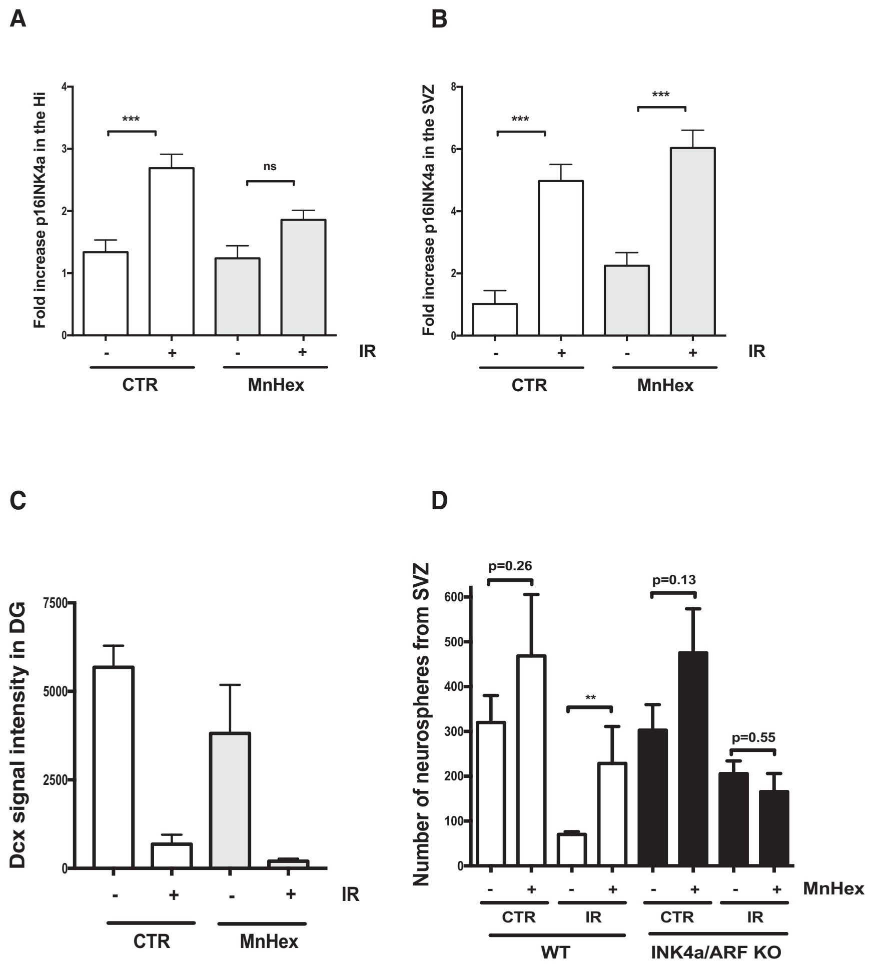

Figure 5. Treatment with a Superoxide Dismutase Mimetic Impacts Neuronal Progenitor Cells Growth *Ex Vivo* but Not *In Vivo*

(A and B) WT mice were treated for eight consecutive weeks with MnHex immediately after exposure to 6 Gy cranial IR. Upon sacrifice, RNA from the hippocampus (A) or SVZ (B) was isolated and p16INK4a expression determined by real-time qPCR.

(C) Quantification of DCX staining was determined in the DG of mice treated or not with MnHex and adjusted to the size of the region on each section.

(D) From the same treated mice, the SVZ regions were dissociated and neurospheres counted. n = 4–10, mice per group. The p values (**$p < 0.01$; ***$p < 0.001$) were obtained by performing a Student's t test.

from p16-3MR mice, presumably because of the weakness of the p16INK4a endogenous promoter and the reporter gene used.

DISCUSSION

Damage induced by radiotherapy is a serious medical concern, with most patients showing long-term treatment-related late effects (Geenen et al., 2007; Krull et al., 2013; Oeffinger et al., 2006). Here, we have identified INK4a/ARF expression as a mechanism responsible for IR-induced long-term loss of neurogenesis in both the DG and SVZ regions.

Previous results by Molofsky et al. (2006) showed that p16INK4a deficiency could partially maintain neurogenesis in the SVZ, but not in the DG, of aged mice, indicating a difference in the sensitivity of these two regions to increase p16INK4a expression. In contrast, we found that the absence of INK4a/ARF expression could partially restore neurogenesis in the DG following exposure to IR. We believe such a discrepancy between the impact of INK4a/ARF expression during aging and following exposure to IR can be explained if, for example, the progenitor cell pool in the DG is fully exhausted during aging but not following exposure to IR—thereby allowing a few residual progenitors to partially restore neurogenesis.

We showed that *ink4a/arf* is transcriptionally activated in irradiated sorted progenitor cells, but unfortunately we were unable to measure its expression at the single-cell level in tissues. This is justified by the unavailability of an antibody capable of detecting endogenous levels of INK4/ARF expression in mouse brain sections and the weakness of the p16INK4a endogenous promoter used in reporter mice. Moreover, consistent with the fact that INK4a/ARF expression is induced several weeks after IR, no increase in neurogenesis was observed in *ink4a/arf-null* mice only 2 weeks after their exposure to IR (Figure S5).

Our results also revealed regional differences, with the DG being more sensitive than the SVZ to IR-induced

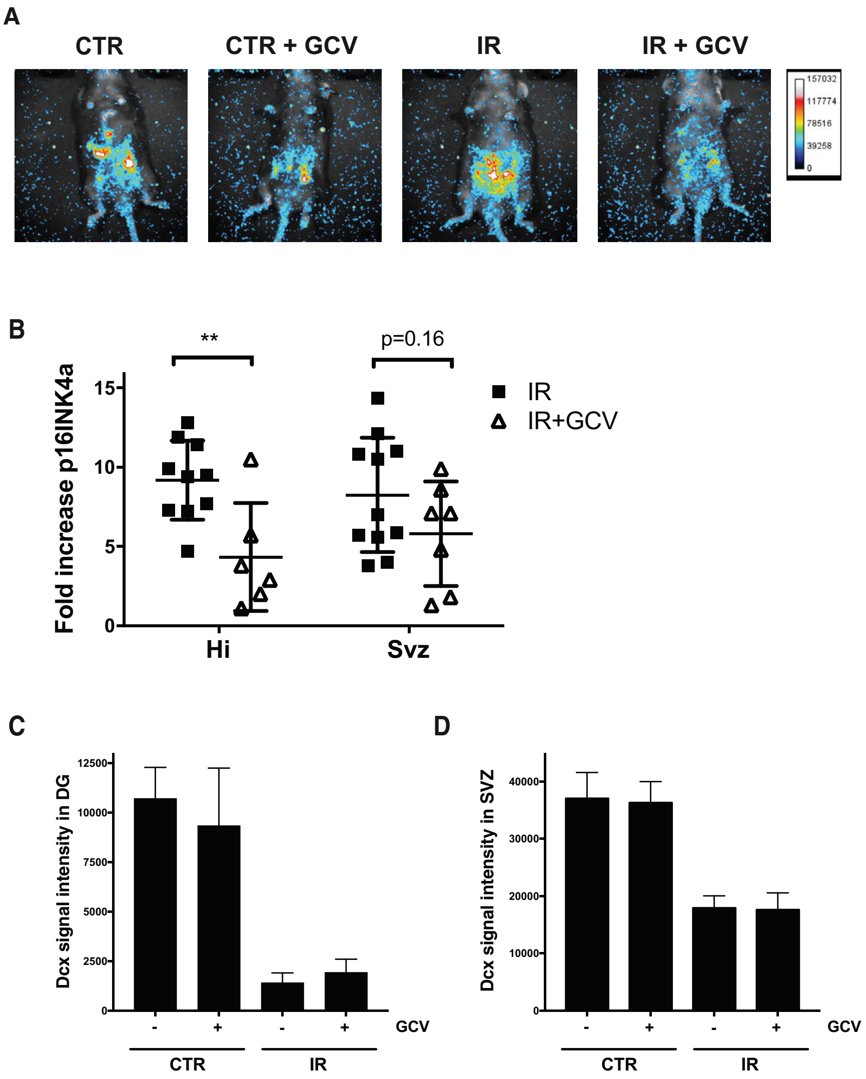

Figure 6. Genetic Elimination of p16INK4a Expressing Cells Does Not Restore Neurogenesis

p16-3MR mice were irradiated or not and 9 weeks later injected with GCV for five consecutive days. Animals were sacrificed 10 days following the last GCV injection.

(A) Mice were placed under anesthesia and luminescence signal detected following the injection of coelenterazine.

(B) RNA was extracted from the DG and SVZ and the expression of p16INK4a determined by real-time qPCR normalized to 18S.

(C and D) Quantification of DCX staining was determined in the DG and SVZ and adjusted to the size of the region on each section. n = 6–11 mice per group with each symbol representing an individual mouse. The p values (**$p < 0.01$) were obtained by performing a Student's t test.

loss of neurogenesis. The reason for such a difference in the sensitivity of these two regions is unknown and may involve different mechanisms. For example, progenitor cells in the DG may be more sensitive to IR-induced INK4a/ARF expression and thus be more prone to replication exhaustion. Alternatively, differences in the microenvironment of these two regions may have played a role, for example, through ROS, which were reported to be elevated in human neural and hematopoietic stem cells following exposure to IR (Acharya et al., 2010; Wang et al., 2010). Indeed, a previous report showed that the administration of MnTE-2-PyP^{5+} (a manganese porphyrin superoxide dismutase mimetic structurally very similar to the MnHex analog we used) can mitigate IR-induced long-term bone marrow suppression in mice and expression of p16INK4a in hematopoietic stem cells (Li et al., 2011). Surprisingly, we found that treatment of irradiated mice with MnHex did not increase neurogenesis in the DG despite allowing for a reduction in p16INK4a expression and higher neurosphere counts. The simplest explanation for this is that the impact of MnHex on p16INK4a expression was too modest. Alternatively, some progenitor cells may have been ready for replication (with presumably low enough p16INK4a), but were maintained in check and unable to replicate because of irreversible damage. Consistent with this hypothesis, we previously showed that irradiated brain tissues maintained an activated DNA damage response for several months following exposure to IR (Fumagalli et al., 2012; Le et al., 2010). Here we found that persistent DNA damage foci accumulate in both neurons and progenitor/stem cells of the irradiated DG region. However, DNA damage accumulated at a lower frequency in newly born neurons from *ink4a/arf* and *p53*-null mice

(Figure 4E), consistent with the higher neurogenesis observed in these mice.

We also consistently observed lower levels of DCX+ cells in the SVZ of *Ink4a/arf-null* mice compared with wild-type or *p53-null* mice (Figure S1A). The reasons for this are unknown but could be explained if young neurons transit more rapidly to a more mature state in the absence of INK4a/ARF. Alternatively, it could be that INK4a/ARF expression is necessary to maintain neurogenesis and/or hamper gliogenesis. Our observation that neurospheres derived from *ink4a/arf*-null mice do not differentiate into neurons *in vitro* supports this hypothesis.

We were surprised to find that the clearance of p16INK4a-expressing cells did not restore, at least partially, neurogenesis (Figure 6C). It is possible that GCV did not sufficiently abrogate p16INK4a expression. Alternatively, progenitor cells themselves may have been cleared by GCV. However, high variability in cell counts within samples from the same groups of mice, presumably because of the harsh protocol used to dissociate cells from the adult brain, prevented us from accurately determining absolute progenitor cell counts. Another possibility is that the microenvironment could have been permanently altered by the irradiation, independently of our capacity to eliminate senescent cells using GCV. In support of this hypothesis, we observed that the conditional deletion of p16INK4a, in previously irradiated mice, also failed to increase neurogenesis, suggesting that loss of neurogenesis is an irreversible process in this context (Figure S6). However, ablation of p16INK4a in specific cell types, either progenitor or niche cells, will be necessary to conclude whether the effect is cell autonomous or not.

Another possibility is that senescent cells restrain neurogenesis through their altered secretion of biologically active molecules, such as growth factors and inflammatory cytokines, referred to as the senescence-associated secretion phenotype or SASP (Coppe et al., 2008; Kuilman et al., 2008; Rodier et al., 2009). However, qPCR analysis performed on hippocampal tissues from wild-type mice collected 8 weeks after irradiation did not reveal aberrant expression of key SASP factors, such as interleukin-6, and the monocyte chemoattractant protein-1 (data not shown). While we cannot rule out that other secreted factors, such as CCL11, may have played a role in inhibiting neurogenesis (Lee et al., 2013; Villeda et al., 2011), our results showing that the elimination of senescent cells using GCV had no effect on neurogenesis suggest the SASP is unlikely to play a major role in IR-induced loss of neurogenesis.

In summary, we believe that treatments looking to preserve stem cell regenerative potential, by limiting INK4a/ARF expression, may help prevent radiation-related loss of neurogenesis. Our results also suggest that the use of senolytic drugs that can reverse aging/senescence will not improve IR-induced loss of neurogenesis in cancer patients.

EXPERIMENTAL PROCEDURES

Mice

8- to 12-week-old female C57BL/6J mice were purchased from Charles River Laboratories (Saint-Constant, Quebec). *Ink4a/arf*-null mice and p16-3MR transgenic mice were bred on site under a Material Transfer Agreement from the National Cancer Institute Mouse Models of Human Cancers Consortium (strain code 01XB1), or from the Buck Institute, respectively. p53 heterozygous mice (B6.129S2-Trp53tm1Tyj/J) were purchased from The Jackson Laboratory. When applicable, mice were allowed to acclimate at least 1 week prior to their use for experimentation. All *in vivo* manipulations were approved by the Comité Institutionnel des Bonnes Pratiques Animales en Recherche of CHU Ste-Justine (protocol no. 579).

Cranial Irradiation and Injection in Mice

Mice were exposed to X-rays at a single sublethal dose of 6 Gy (1 Gy/min) using a Faxitron CP-160. Mice were anesthetized during the procedure and only the head and neck were exposed to radiation, with the remainder of the body being shielded by a lead cover; the exception being p16-3MR mice that received total body irradiation. For neurogenesis studies, mice were injected intraperitoneally with BrdU (Sigma) at a dose of 50 mg/kg once a day for 7–10 consecutive days. Porphyrin-based potent superoxide dismutase mimetic (Mn(III) *meso*-tetrakis-(n-hexylpyridinium-2-yl) porphyrin, MnTnHex-2-PyP^{5+} (MnHex) was administered at a dose of 450 μg/kg/day subcutaneously for eight consecutive weeks using Alzet pumps (model 1004 loaded with MnHex diluted in saline at a concentration of 3.2 mM). NAC was given in drinking water for eight consecutive weeks at a dose of 1,200 mg/kg/day. Both MnHex and NAC treatments started immediately after exposure of mice to 6 Gy cranial IR. GCV was injected intraperitoneally at a dose of 25 mg/kg for five consecutive days starting 9 weeks post IR. Luminescence was quantified using a HNü EMCCD camera (Nüvü Camēras) housed in an *in vivo* epi-fluorescence and trans-fluorescence imaging system from Labeo Technologies, 15 min following the intraperitoneal injection of 200 μg of coelenterazine (NanoLight Technology) diluted in PBS.

Tissue Preparation

For DCX staining, mice were anesthetized using intraperitoneal injection of pentobarbital at a dose of 65 mg/kg and then perfused transcardially, first with 20 mL of saline containing heparine (10 U/mL), followed by 20 mL of 3.7% formaldehyde. The brain was removed and fixed in formaldehyde overnight at 4°C. Brains were then equilibrated in 40% sucrose overnight at 4°C and embedded in OCT compound and stored at −80°C until use. Coronal sections of 40 μm were obtained and stored at −20°C in an antifreeze solution (20% glycerol and 30% ethylene glycol in PBS). For TUNEL and CD68 stainings, mouse brains were frozen directly on dry ice. Cryosections of 10 μm were mounted on microscope glass slides previously coated with 1% gelatin and

0.05% chromium alum, dried at room temperature, and then stored at −80°C until use.

Immunofluorescence

All sections were permeabilized and blocked as described previously (Le et al., 2010). For BrdU immunostaining, the sections were subsequently treated with 0.5 N HCl at 37°C for 30 min to denature the DNA and then neutralized with 0.1 M borate buffer (pH 8.5) at room temperature for 10 min. Primary antibodies used in this study were: DCX goat anti-human (clone C-18 from Santa Cruz Biotechnology at a dilution of 1:500), mouse monoclonal BrdU (clone B44 from BD Biosciences at a dilution of 1:250), rat anti-mouse CD68 (clone FA-11AbD from Serotec at a dilution of 1:500), SOX-2 mouse IgG2a (clone 245610 from R&D Systems, cat. no. MAB2018-SP at a dilution of 1:250), 53BP1 rabbit polyclonal (Novus Biologicals, cat. no. NB100-304 at a dilution of 1:500). Apoptosis was measured using the In Situ Cell Death Detection Kit, Fluorescein (TdT-mediated dUTP Nick End Labeling) according to the manufacturer's instructions (Roche). Wide-field fluorescence images were obtained using an Olympus BX51 epifluorescence microscope. Confocal images were obtained using an Ultraview Vox spinning disc confocal system (PerkinElmer), employing a CSU-X1 scanning unit (Yokogawa) and an ORCA-R2 CCD camera (Hamamatsu) fitted to a Leica DMI6000B inverted microscope, using Plan Apo 40× (0.85 NA) and 10× objectives. DCX signal intensity was quantified using Image-Pro. DNA damage confocal images were obtained using a Leica microsystems TSC SP8 HyVolution microscope.

Neurosphere Formation Assay and Differentiation

Mice were euthanized and the cell layers that form the ventricles, mainly the SVZ, were quickly isolated in dissecting buffer containing: 125 mM NaCl, 50 mM KCl, 25 mM $NaHCO_3$, 1.25 mM NaH_2PO_4 (monobasic), 20 mM glucose, 0.1 mM $CaCl_2$, and 3.2 mM $MgCl_2$. Dissected tissue was then transferred into an enzyme mix solution containing 0.13% trypsin (necessary with adult's brains), 0.067% type 1S hyaluronidase, and 0.013% kynurenic acid (all purchased from Sigma). The digestion was done at 37°C for 30 min. Cells were then centrifuged and the cell pellet washed for 10 min at 37°C in dissecting solution supplemented with 0.05% trypsin inhibitor and 0.5% BSA (both purchased from Sigma). Cells were then centrifuged and filtered through a 40-μm cell strainer in dissecting solution. Cells were then resuspended in DMEM/F12 (Invitrogen) supplemented with 2 mM L-glutamine, 25 μM L-glutamate, 0.6% glucose, 20 ng/mL mouse epidermal growth factor (mEGF) (Sigma), 10 ng/mL basic fibroblast growth factor (bFGF) (Antigenix America), B27 supplement, and 1× N-2 supplement (Invitrogen Life Technologies). Cells (bulk) were then plated at a ratio of 2×10^5 cells/well onto an ultralow attachment 96-well plate. Neurosphere were counted manually using an inverted microscope 10–14 days later. Secondary neurospheres counts were determined by the dissociation of primary neurospheres and by plating cells at a density of 2×10^4 cells onto an ultralow attachment 96-well plate. Neurospheres were counted manually using an inverted microscope 7 days later. To measure their differentiation potential, secondary neurospheres were dissociated and first expanded for 7 days in NeuroCult basal medium with NeuroCult proliferation supplement (STEMCELL Technologies), together with mEGF (20 mg/mL, Sigma), bFGF (10 ng/mL, Antigenix America), and heparin (2 μg/mL, Sigma). Cells were then transferred on Matrigel-coated glass coverslips and differentiated using NeuroCult differentiation supplement as recommended by the manufacturer. After 7–10 days, cells grown on coverslips were fixed in 4% paraformaldehyde (in PBS [pH 7.4]) and processed for the detection of NeuN (mouse immunoglobulin G [IgG] clone A60 from Millipore, cat. no. MAB377 at a dilution of 1:250), glial fibrillary acidic protein (rabbit IgG from DAKO, cat. no. Z033429-2 at a dilution of 1:1,500) and the oligodendrocyte marker O4 (mouse IgM clone O4 from R&D Systems, cat. no. MAB1326-SP at the dilution of 1:300).

RNA Isolation and qPCR

Excised tissues sliced into ~2-mm^2 pieces and preserved in RNA Later (QIAGEN) were mechanically dissociated in 500 μL Qiazol lysis reagent (QIAGEN) using a homogenizer (OMNI International), and total RNA was extracted using the RNeasy Lipid Tissue Mini Kit (QIAGEN). Alternatively, hippocampus and SVZ regions were dissected and dissociated using the adult brain dissociation kit from Miltenyi Biotec and the gentleMACS Octo Dissociator with heaters. Cells were then sorted on a FACSDiva 8.0.1 (BD Biosciences) directly into lysis solution (QIAzol) using the following antibodies as reported (Daynac et al., 2013): fluorescein isothiocyanate mouse LEX/CD15 (BD Biosciences, cat. no. 560127, clone MC480), PE rat CD24 (BD Biosciences, cat. no. 553262, clone M1/69), Alexa 647-conjugated EGF (Molecular Probes E35351). One μg or less of RNA was then reverse-transcribed using the QuantiTect Reverse Transcription Kit (QIAGEN). Quantitative differences in gene expression were determined by real-time qPCR using SYBR Green PCR Master Mix (Bio-Rad) and a spectrofluorometric thermal cycler (Mx3000P from Stratagene). Values are presented as the ratio of target mRNA to 18S rRNA obtained using the relative standard curve method of calculation. Primers sequences were described previously (Le et al., 2010).

Statistical Analysis

Statistical analyses were performed with the GraphPad Prism 7.0 software.

SUPPLEMENTAL INFORMATION

Supplemental Information includes six figures and can be found with this article online at https://doi.org/10.1016/j.stemcr.2018.03.025.

AUTHOR CONTRIBUTIONS

O.L., collection and/or assembly of data and data analysis and interpretation. G.B., provision of study material and interpretation of the data. L. P., collection and/or assembly of data. I.B.-H., provision of study material. G.H., collection and/or assembly of data. C.B., data analysis and interpretation, manuscript writing, and financial support.

ACKNOWLEDGMENTS

We would like to thank Denise Carrier for her help with mice. This work was supported by a grant from the Canadian Institute of

Health Research (MOP-102709) (C.B.) and a Canadian Foundation for Innovation infrastructure award (G.H.). C.B. and G.H. are supported by senior awards from the FRQS. G.H. also thanks the Cole Foundation for a transition award.

Received: April 27, 2017
Revised: March 28, 2018
Accepted: March 29, 2018
Published: April 26, 2018

REFERENCES

Acharya, M.M., Lan, M.L., Kan, V.H., Patel, N.H., Giedzinski, E., Tseng, B.P., and Limoli, C.L. (2010). Consequences of ionizing radiation-induced damage in human neural stem cells. Free Radic. Biol. Med. *49*, 1846–1855.

Baker, D.J., Childs, B.G., Durik, M., Wijers, M.E., Sieben, C.J., Zhong, J., Saltness, R.A., Jeganathan, K.B., Verzosa, G.C., Pezeshki, A., et al. (2016). Naturally occurring p16(Ink4a)-positive cells shorten healthy lifespan. Nature *530*, 184–189.

Baker, D.J., Weaver, R.L., and van Deursen, J.M. (2013). p21 both attenuates and drives senescence and aging in BubR1 progeroid mice. Cell Rep. *3*, 1164–1174.

Batinic-Haberle, I., Tovmasyan, A., Roberts, E.R., Vujaskovic, Z., Leong, K.W., and Spasojevic, I. (2014). SOD therapeutics: latest insights into their structure-activity relationships and impact on the cellular redox-based signaling pathways. Antioxid. Redox Signal. *20*, 2372–2415.

Batinic-Haberle, I., Tovmasyan, A., and Spasojevic, I. (2015). An educational overview of the chemistry, biochemistry and therapeutic aspects of Mn porphyrins–from superoxide dismutation to H2O2-driven pathways. Redox Biol. *5*, 43–65.

Coppe, J.P., Patil, C.K., Rodier, F., Sun, Y., Munoz, D.P., Goldstein, J., Nelson, P.S., Desprez, P.Y., and Campisi, J. (2008). Senescence-associated secretory phenotypes reveal cell-nonautonomous functions of oncogenic RAS and the p53 tumor suppressor. PLoS Biol. *6*, 2853–2868.

Daynac, M., Chicheportiche, A., Pineda, J.R., Gauthier, L.R., Boussin, F.D., and Mouthon, M.A. (2013). Quiescent neural stem cells exit dormancy upon alteration of GABAAR signaling following radiation damage. Stem Cell Res. *11*, 516–528.

Demaria, M., Ohtani, N., Youssef, S.A., Rodier, F., Toussaint, W., Mitchell, J.R., Laberge, R.M., Vijg, J., Van Steeg, H., Dolle, M.E., et al. (2014). An essential role for senescent cells in optimal wound healing through secretion of PDGF-AA. Dev. Cell *31*, 722–733.

Despars, G., Carbonneau, C.L., Bardeau, P., Coutu, D.L., and Beausejour, C.M. (2013). Loss of the osteogenic differentiation potential during senescence is limited to bone progenitor cells and is dependent on p53. PLoS One *8*, e73206.

Ekdahl, C.T., Claasen, J.H., Bonde, S., Kokaia, Z., and Lindvall, O. (2003). Inflammation is detrimental for neurogenesis in adult brain. Proc. Natl. Acad. Sci. USA *100*, 13632–13637.

Fumagalli, M., Rossiello, F., Clerici, M., Barozzi, S., Cittaro, D., Kaplunov, J.M., Bucci, G., Dobreva, M., Matti, V., Beausejour, C.M., et al. (2012). Telomeric DNA damage is irreparable and causes persistent DNA-damage-response activation. Nat. Cell Biol. *14*, 355–365.

Geenen, M.M., Cardous-Ubbink, M.C., Kremer, L.C., van den Bos, C., van der Pal, H.J., Heinen, R.C., Jaspers, M.W., Koning, C.C., Oldenburger, F., Langeveld, N.E., et al. (2007). Medical assessment of adverse health outcomes in long-term survivors of childhood cancer. JAMA *297*, 2705–2715.

Giustarini, D., Milzani, A., Dalle-Donne, I., Tsikas, D., and Rossi, R. (2012). N-Acetylcysteine ethyl ester (NACET): a novel lipophilic cell-permeable cysteine derivative with an unusual pharmacokinetic feature and remarkable antioxidant potential. Biochem. Pharmacol. *84*, 1522–1533.

Hellstrom, N.A., Bjork-Eriksson, T., Blomgren, K., and Kuhn, H.G. (2009). Differential recovery of neural stem cells in the subventricular zone and dentate gyrus after ionizing radiation. Stem Cells *27*, 634–641.

Hellstrom, N.A., Lindberg, O.R., Stahlberg, A., Swanpalmer, J., Pekny, M., Blomgren, K., and Kuhn, H.G. (2011). Unique gene expression patterns indicate microglial contribution to neural stem cell recovery following irradiation. Mol. Cell Neurosci. *46*, 710–719.

Ito, K., Hirao, A., Arai, F., Matsuoka, S., Takubo, K., Hamaguchi, I., Nomiyama, K., Hosokawa, K., Sakurada, K., Nakagata, N., et al. (2004). Regulation of oxidative stress by ATM is required for self-renewal of haematopoietic stem cells. Nature *431*, 997–1002.

Ito, K., Hirao, A., Arai, F., Takubo, K., Matsuoka, S., Miyamoto, K., Ohmura, M., Naka, K., Hosokawa, K., Ikeda, Y., et al. (2006). Reactive oxygen species act through p38 MAPK to limit the lifespan of hematopoietic stem cells. Nat. Med. *12*, 446–451.

Krishnamurthy, J., Ramsey, M.R., Ligon, K.L., Torrice, C., Koh, A., Bonner-Weir, S., and Sharpless, N.E. (2006). p16INK4a induces an age-dependent decline in islet regenerative potential. Nature *443*, 453–457.

Krishnamurthy, J., Torrice, C., Ramsey, M.R., Kovalev, G.I., Al-Regaiey, K., Su, L., and Sharpless, N.E. (2004). Ink4a/Arf expression is a biomarker of aging. J. Clin. Invest. *114*, 1299–1307.

Krull, K.R., Brinkman, T.M., Li, C., Armstrong, G.T., Ness, K.K., Srivastava, D.K., Gurney, J.G., Kimberg, C., Krasin, M.J., Pui, C.H., et al. (2013). Neurocognitive outcomes decades after treatment for childhood acute lymphoblastic leukemia: a report from the St Jude lifetime cohort study. J. Clin. Oncol. *31*, 4407–4415.

Kuilman, T., Michaloglou, C., Vredeveld, L.C., Douma, S., van Doorn, R., Desmet, C.J., Aarden, L.A., Mooi, W.J., and Peeper, D.S. (2008). Oncogene-induced senescence relayed by an interleukin-dependent inflammatory network. Cell *133*, 1019–1031.

Le, O.N., Rodier, F., Fontaine, F., Coppe, J.P., Campisi, J., DeGregori, J., Laverdiere, C., Kokta, V., Haddad, E., and Beausejour, C.M. (2010). Ionizing radiation-induced long-term expression of senescence markers in mice is independent of p53 and immune status. Aging Cell *9*, 398–409.

Lee, S.W., Haditsch, U., Cord, B.J., Guzman, R., Kim, S.J., Boettcher, C., Priller, J., Ormerod, B.K., and Palmer, T.D. (2013). Absence of CCL2 is sufficient to restore hippocampal neurogenesis following cranial irradiation. Brain Behav. Immun. *30*, 33–44.

Li, H., Wang, Y., Pazhanisamy, S.K., Shao, L., Batinic-Haberle, I., Meng, A., and Zhou, D. (2011). Mn(III) meso-tetrakis-(N-ethylpyridinium-2-yl) porphyrin mitigates total body irradiation-induced long-term bone marrow suppression. Free Radic. Biol. Med. *51*, 30–37.

Limoli, C.L., Giedzinski, E., Rola, R., Otsuka, S., Palmer, T.D., and Fike, J.R. (2004). Radiation response of neural precursor cells: linking cellular sensitivity to cell cycle checkpoints, apoptosis and oxidative stress. Radiat. Res. *161*, 17–27.

Lu, F.G., and Wong, C.S. (2007). Time-dependent neurosphere-forming ability of adult rat spinal cord after irradiation. Radiat. Res. *168*, 453–461.

Marcoux, S., Le, O.N., Langlois-Pelletier, C., Laverdiere, C., Hatami, A., Robaey, P., and Beausejour, C.M. (2013). Expression of the senescence marker p16INK4a in skin biopsies of acute lymphoblastic leukemia survivors: a pilot study. Radiat. Oncol. *8*, 252.

Mizumatsu, S., Monje, M.L., Morhardt, D.R., Rola, R., Palmer, T.D., and Fike, J.R. (2003). Extreme sensitivity of adult neurogenesis to low doses of X-irradiation. Cancer Res. *63*, 4021–4027.

Molofsky, A.V., Slutsky, S.G., Joseph, N.M., He, S., Pardal, R., Krishnamurthy, J., Sharpless, N.E., and Morrison, S.J. (2006). Increasing p16INK4a expression decreases forebrain progenitors and neurogenesis during ageing. Nature *443*, 448–452.

Monje, M., and Dietrich, J. (2012). Cognitive side effects of cancer therapy demonstrate a functional role for adult neurogenesis. Behav. Brain Res. *227*, 376–379.

Monje, M.L., Mizumatsu, S., Fike, J.R., and Palmer, T.D. (2002). Irradiation induces neural precursor-cell dysfunction. Nat. Med. *8*, 955–962.

Monje, M.L., Toda, H., and Palmer, T.D. (2003). Inflammatory blockade restores adult hippocampal neurogenesis. Science *302*, 1760–1765.

Moravan, M.J., Olschowka, J.A., Williams, J.P., and O'Banion, M.K. (2011). Cranial irradiation leads to acute and persistent neuroinflammation with delayed increases in T-cell infiltration and CD11c expression in C57BL/6 mouse brain. Radiat. Res. *176*, 459–473.

Oeffinger, K.C., Mertens, A.C., Sklar, C.A., Kawashima, T., Hudson, M.M., Meadows, A.T., Friedman, D.L., Marina, N., Hobbie, W., Kadan-Lottick, N.S., et al. (2006). Chronic health conditions in adult survivors of childhood cancer. N. Engl. J. Med. *355*, 1572–1582.

Oubaha, M., Miloudi, K., Dejda, A., Guber, V., Mawambo, G., Germain, M.A., Bourdel, G., Popovic, N., Rezende, F.A., Kaufman, R.J., et al. (2016). Senescence-associated secretory phenotype contributes to pathological angiogenesis in retinopathy. Sci. Transl. Med. *8*, 362ra144.

Rao, A.A., Ye, H., Decker, P.A., Howe, C.L., and Wetmore, C. (2011). Therapeutic doses of cranial irradiation induce hippocampus-dependent cognitive deficits in young mice. J. Neurooncol. *105*, 191–198.

Robison, L.L., and Hudson, M.M. (2014). Survivors of childhood and adolescent cancer: life-long risks and responsibilities. Nat. Rev. Cancer *14*, 61–70.

Rodier, F., Coppe, J.P., Patil, C.K., Hoeijmakers, W.A., Munoz, D.P., Raza, S.R., Freund, A., Campeau, E., Davalos, A.R., and Campisi, J. (2009). Persistent DNA damage signalling triggers senescence-associated inflammatory cytokine secretion. Nat. Cell Biol. *11*, 973–979.

Rola, R., Raber, J., Rizk, A., Otsuka, S., VandenBerg, S.R., Morhardt, D.R., and Fike, J.R. (2004). Radiation-induced impairment of hippocampal neurogenesis is associated with cognitive deficits in young mice. Exp. Neurol. *188*, 316–330.

Schafer, M.J., White, T.A., Iijima, K., Haak, A.J., Ligresti, G., Atkinson, E.J., Oberg, A.L., Birch, J., Salmonowicz, H., Zhu, Y., et al. (2017). Cellular senescence mediates fibrotic pulmonary disease. Nat. Commun. *8*, 14532.

Sharpless, N.E., Bardeesy, N., Lee, K.H., Carrasco, D., Castrillon, D.H., Aguirre, A.J., Wu, E.A., Horner, J.W., and DePinho, R.A. (2001). Loss of p16Ink4a with retention of p19Arf predisposes mice to tumorigenesis. Nature *413*, 86–91.

Tada, E., Yang, C., Gobbel, G.T., Lamborn, K.R., and Fike, J.R. (1999). Long-term impairment of subependymal repopulation following damage by ionizing irradiation. Exp. Neurol. *160*, 66–77.

van Deursen, J.M. (2014). The role of senescent cells in ageing. Nature *509*, 439–446.

Villeda, S.A., Luo, J., Mosher, K.I., Zou, B., Britschgi, M., Bieri, G., Stan, T.M., Fainberg, N., Ding, Z., Eggel, A., et al. (2011). The ageing systemic milieu negatively regulates neurogenesis and cognitive function. Nature *477*, 90–94.

Wang, Y., Liu, L., Pazhanisamy, S.K., Li, H., Meng, A., and Zhou, D. (2010). Total body irradiation causes residual bone marrow injury by induction of persistent oxidative stress in murine hematopoietic stem cells. Free Radic. Biol. Med. *48*, 348–356.

Wang, Y., Schulte, B.A., LaRue, A.C., Ogawa, M., and Zhou, D. (2006). Total body irradiation selectively induces murine hematopoietic stem cell senescence. Blood *107*, 358–366.

Weitner, T., Kos, I., Sheng, H., Tovmasyan, A., Reboucas, J.S., Fan, P., Warner, D.S., Vujaskovic, Z., Batinic-Haberle, I., and Spasojevic, I. (2013). Comprehensive pharmacokinetic studies and oral bioavailability of two Mn porphyrin-based SOD mimics, MnTE-2-PyP5+ and MnTnHex-2-PyP5+. Free Radic. Biol. Med. *58*, 73–80.

Wojtowicz, J.M. (2006). Irradiation as an experimental tool in studies of adult neurogenesis. Hippocampus *16*, 261–266.

Zou, Y., Corniola, R., Leu, D., Khan, A., Sahbaie, P., Chakraborti, A., Clark, D.J., Fike, J.R., and Huang, T.T. (2012). Extracellular superoxide dismutase is important for hippocampal neurogenesis and preservation of cognitive functions after irradiation. Proc. Natl. Acad. Sci. USA *109*, 21522–21527.

Stem Cell Reports, Volume *10*

Supplemental Information

INK4a/ARF Expression Impairs Neurogenesis in the Brain of Irradiated Mice

Oanh Le, Lina Palacio, Gilbert Bernier, Ines Batinic-Haberle, Gilles Hickson, and Christian Beauséjour

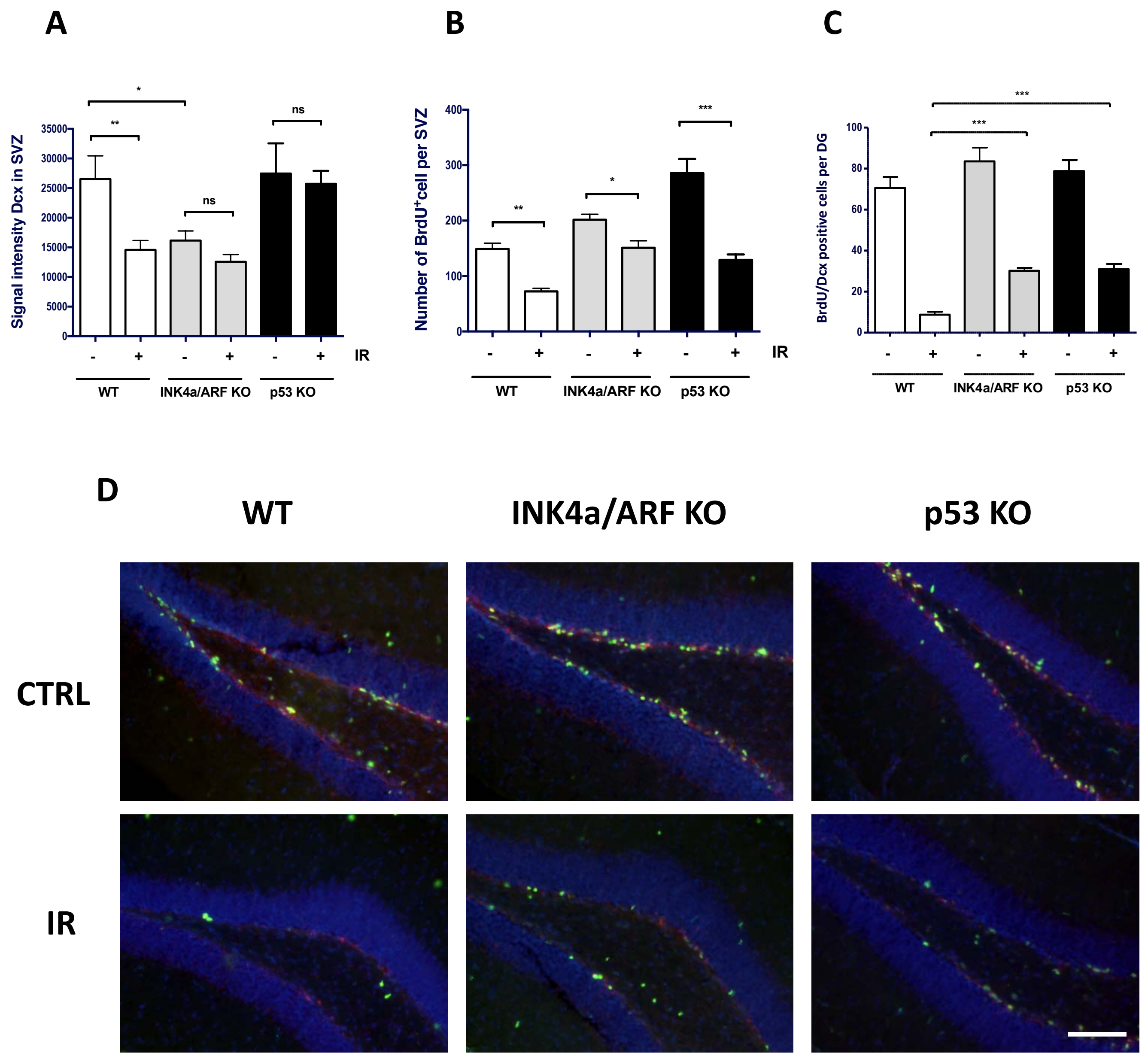

Figure S1. Neurogenesis recovery after IR in absence of INK4a/ARF or p53 expression. (A) Wild-type (WT), *ink4a/arf*-null (INK4a/ARF KO) and *p53*-null (p53 KO) mice were irradiated or not at a dose of 6 Gy and quantification of Dcx signal intensity in the SVZ determined **(B)** Numbers of BrdU positive cells detected in SVZ. **(C)** BrdU incorporation mostly occurs in Dcx positive cells independently of mouse genotypes. Mice were irradiated or not at a dose of 6 Gy and quantification of double positive Dcx/BrdU cells in the DG. **(D)** Representative images showing BrdU incorpotation in Dcx positive cells in the DG. Scale bar = 100 microns. Average from n= 3-6 sections from at least 3 different mice were used. p values were obtained by performing a non parametric ANOVA (Kruskal-Walli) test (***p<0.001, **p<0.01, *p<0.05). ns= no significant difference was observed.

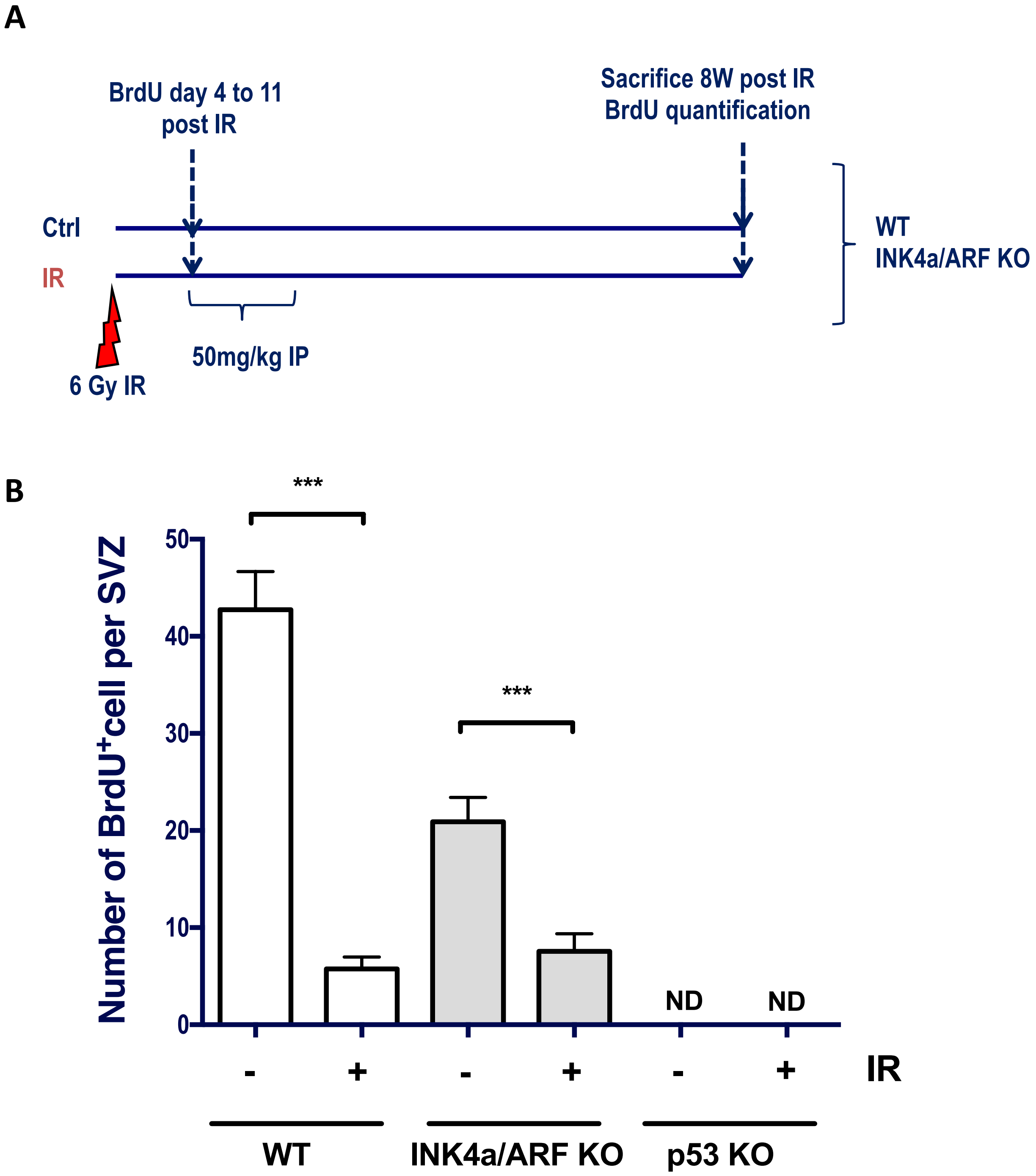

Figure S2. The proportion of BrdU retaining cell long term after IR in WT and INK4a/ARF KO mice. (A) Schematic of the experiment. **(B)** Wild-type (WT) and *ink4a/arf*-null (INK4a/ARF KO) mice were irradiated (6 Gy) and injected intraperitoneally (IP) with BrdU at a dose of 50 mg/kg once a day for 7 days starting on day 4 post exposure to IR. 8 weeks later, mice were sacrificed and the number of BrdU positive cells remaining in the SVZ counted. Shown is the average number of cells per field. n= 6 sections from at least 3 different mice was used. ***p<0.001. nd = not determined.

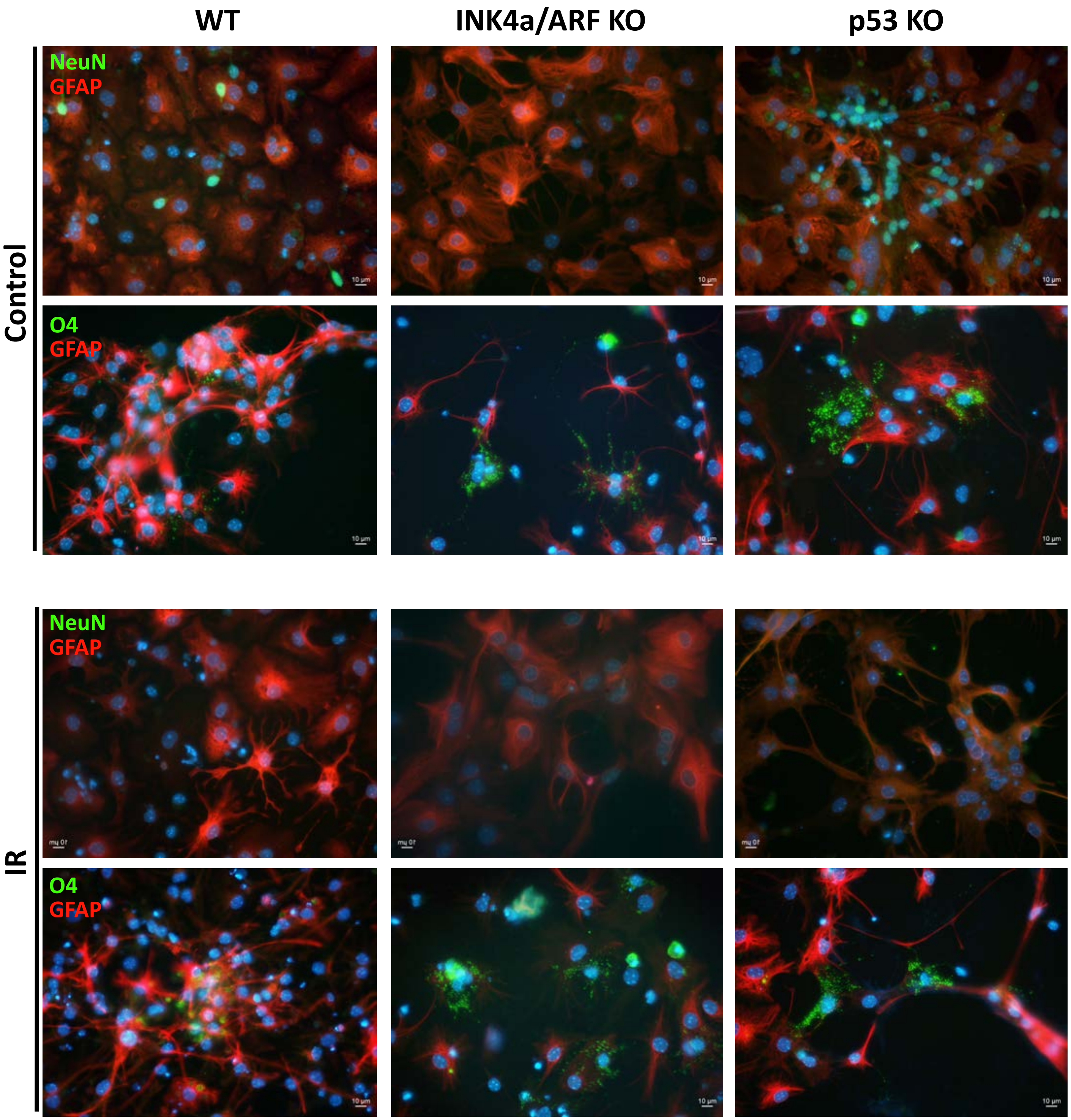

Figure S3. Differentiation potential of secondary neurospheres. Secondary neurospheres from control or irradiated (IR) wild-type (WT), *ink4a/arf*-null (INK4a/ARF KO) and *p53*-null (p53 KO) mice were dissociated, expanded and differentiated into neurons (NeuN in green, upper panel), astrocytes (GFAP in red) or oligodendrocyte (O4 in green, lower panel). Representative images of 2 independent experiments are shown. Nuclei were stained with DAPI (in blue).

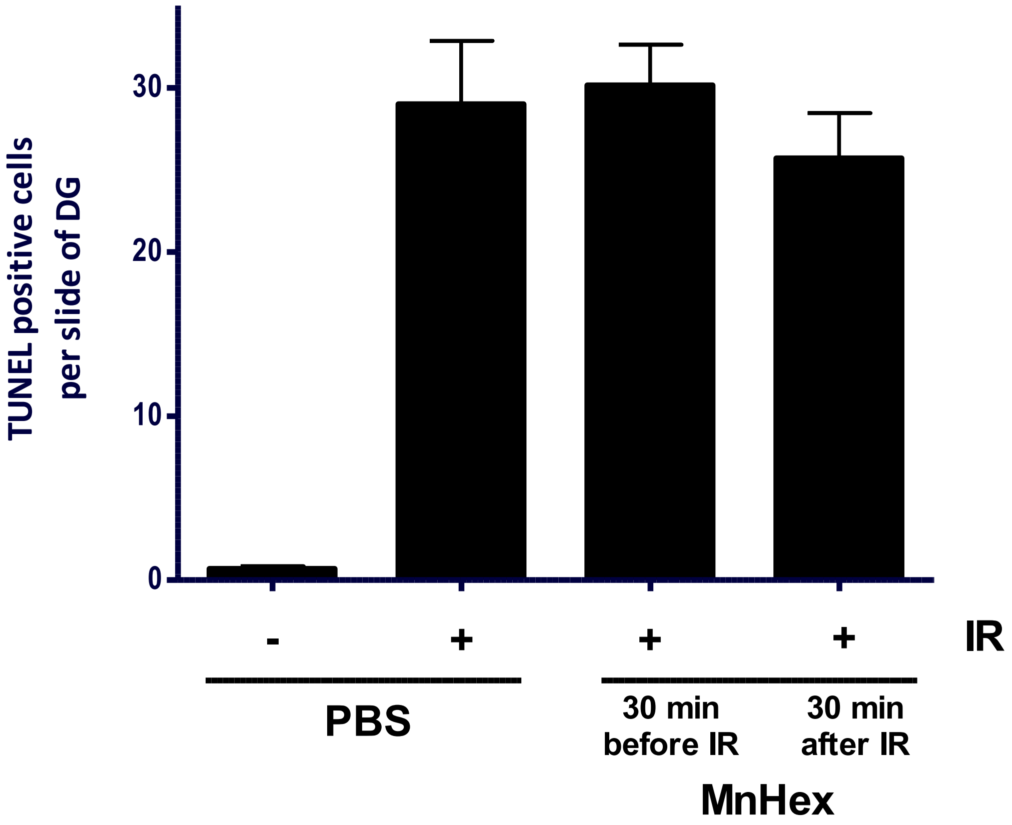

Figure S4. Injection of MnHex does not prevent induction of apoptosis in the irradiated brain. Quantification of the number of apoptotic cells, as detected by TUNEL, in the DG 6 hours following exposure of wild-type (WT) mice injected subcutaneously or not with a single dose of 450 ug/kg of MnHex 30 minutes before or after 6 Gy cranial IR. Indicated is the average number of apoptotic cells per DG section +/- SEM n=4 mice per group.

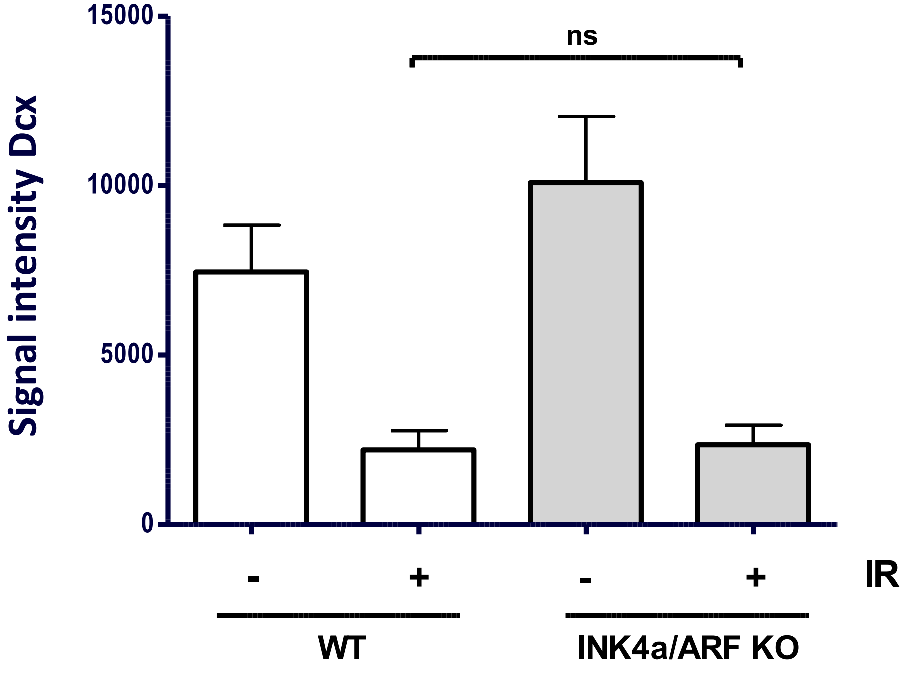

Figure S5. Absence of INK4a/ARF expression does not favors neurogenesis in the DG short term following exposure to IR. Wild-type (WT) and *ink4a/arf*-null (INK4a/ARF KO) mice received or not cranial irradiation at a dose of 6 Gy and quantification of Dcx signal intensity was determined in the DG two weeks after IR. Shown is the average +/- SEM. n= 4 mice per group. NS, no significant difference was observed.

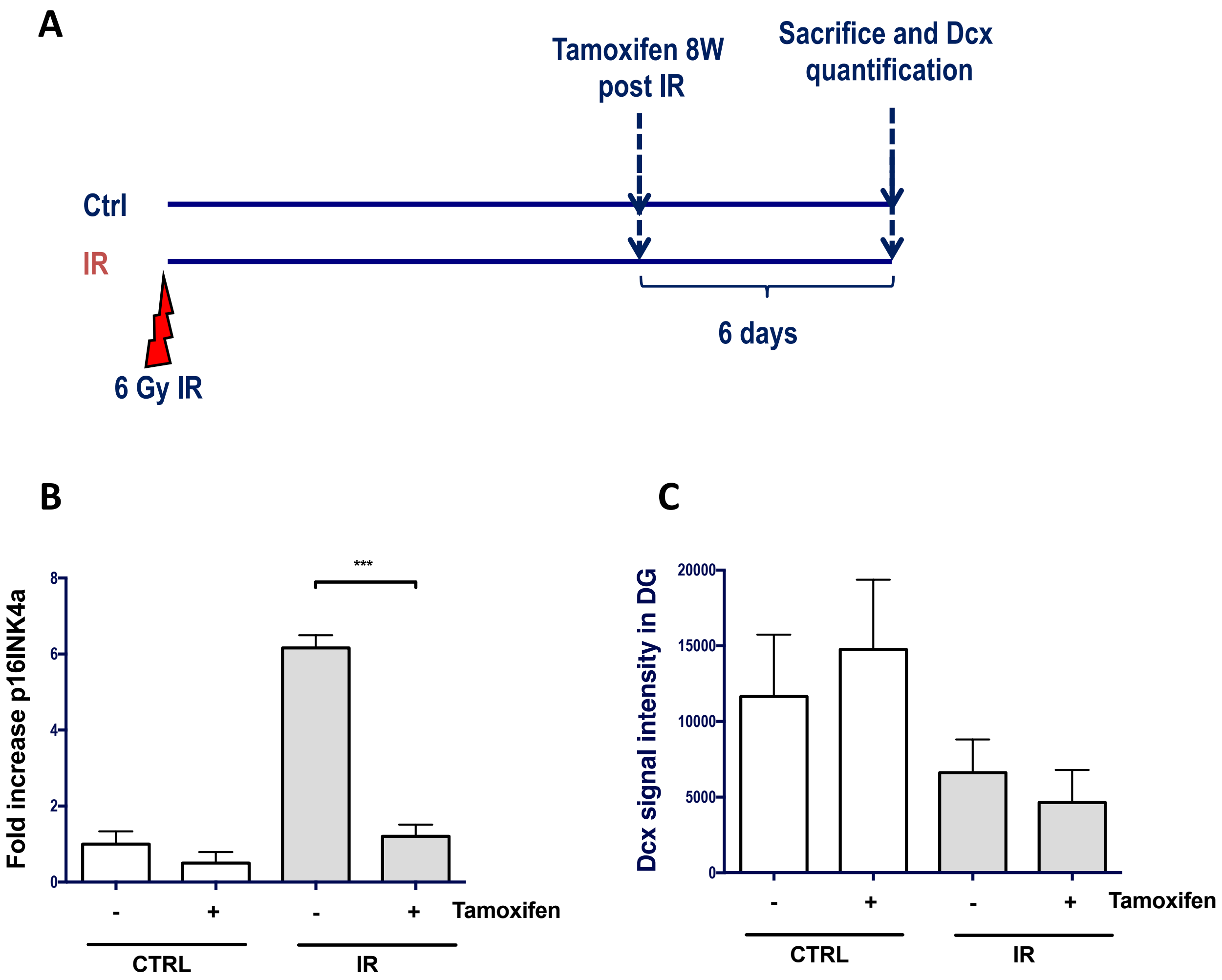

Figure S6. Conditional deletion of p16INK4a does not increase neurogenesis in the DG following exposure to IR. We used conditional *p16INK4a*-null mice in which p16INK4a expression is lost upon the injection of tamoxifen (Cre-ERT^2 recombinase under the human ubiquitin C promoter). **(A)** Schematic of the experiment. 8 weeks after irradiation, mice were treated or not with tamoxifen at a dose of 200 mg/kg (diluted in a mixture 1:50 of ethanol and corn oil respectively – see Palacio et al. Oncogene 2016) by gavage for 5 consecutive days. Mice were sacrificed on day 6 following the first injection of tamoxifen. **(B)** RNA was extracted from the brain and expression of p16INK4a determined by quantitative real-time PCR and normalized to 18S. **(C)** Quantification of Dcx staining was determined in the DG and signal intensity adjusted to the size of the DG on each section. Shown is the average +/- SEM from n= 10-12 sections collected from 4 different mice per group. ***p<0.001.
